# Endothelial CYB5R1 is a Coenzyme Q reductase that suppresses ferroptosis and atherosclerosis

**DOI:** 10.21203/rs.3.rs-7603489/v1

**Published:** 2025-09-29

**Authors:** Robert Hall, Svetlana Samovich, Mate Katona, Shuai Yuan, Megan P. Miller, Scott Hahn, Rolando Cuevas, Fei Chang, Sonia R. Salvatore, Maximiliano Vazquez, Ahssan Sekandari, Cynthia St. Hilaire, Marco Fazzari, Bruce A. Freeman, Francisco Jose Schopfer, Valerian E. Kagan, Hulya Bayir, Adam C. Straub

**Affiliations:** 1 Department of Pharmacology and Chemical Biology, University of Pittsburgh, Pittsburgh, PA; 2 Heart, Lung, Blood and Vascular Medicine Institute, University of Pittsburgh, Pittsburgh, PA; 3 Department of Pediatrics, Columbia University, New York, NY, USA.; 4 Center for Microvascular Research, University of Pittsburgh, Pittsburgh, PA; 5 Department of Environmental Health, University of Pittsburgh, Pittsburgh, PA; 6 Departments of Medicine, Division of Cardiology, Cardiothoracic Surgery and Bioengineering, University of Pittsburgh, PA.

**Keywords:** CYB5R1, ferroptosis, redox, lipid oxidation, CoQ, endothelial cell, atherosclerosis

## Abstract

Spatial-temporal coordination of oxidoreductase substrate specificity and turnover regulates redox-mediated signaling, shaping physiological and pathological outcomes. Here, we reveal novel actions of cytochrome b5 reductase 1 (CYB5R1) when localized to the outer mitochondrial membrane of endothelial cells. Specifically, CYB5R1 functions as a Coenzyme Q (CoQ)–dependent redox cycler, protecting against iron-dependent lipid oxidation or ferroptosis. CYB5R1 catalyzes CoQ redox cycling via electron transfer reactions that suppresses both lipid hydroperoxide accumulation and ferroptosis. CoQ-insufficiency or disruption of CYB5R1-CoQ coupling impairs these reactions, leading to elevated hydrogen peroxide production and initiating a ferroptotic cascade. Ferroptosis plays a pathogenic role in atherogenesis, and we report that both global and endothelial-specific CYB5R1 knockout significantly exacerbate plaque formation. Through a rational chemical library design and screening, we synthesized and tested CP50, a quinone–nitroalkene hybrid that upregulates CYB5R1, prevents glutathione peroxidase-4 (GPX4) degradation, limits lipid oxidation, confers potent anti-ferroptotic activity and in a murine model, profoundly inhibit atherogenesis. These findings a) establish CYB5R1 as a novel mitochondrial “redox rheostat” that governs endothelial ferroptotic susceptibility through substrate redox regulation and b) reveals a safe small molecule therapeutic strategy that can impact a broad range of diseases.

Atherosclerosis is the leading chronic inflammatory disease of the arteries, contributing to nearly 50% of all deaths in western societies^1^. While dysregulated reduction-oxidation (redox) metabolism, particularly lipid oxidation, has been implicated in endothelial (EC) dysfunction and cell death, the precise molecular mechanisms underlying atherogenesis remain incompletely understood^2,3^. Among different types of regulated cell death, ferroptosis has been identified to play a significant role in the pathogenesis of a multitude of chronic diseases^4–6^, including atherosclerosis, due to strong linkages with redox imbalance and iron-dependent phospholipid oxidation^4,7,8^. The regulatory role of redox reductases, particularly NADPH-dependent ferroptosis suppressing protein 1 (FSP-1), in ferroptosis has been documented for several types of cells and tissues^9–11^, but not in endothelial cells in the context of atherosclerosis.

The evolutionarily-conserved cytochrome b5 reductases (CYB5R) play a pivotal role in maintaining redox balance by facilitating electron transfer to essential redox acceptors, such as heme and Coenzyme Q (CoQ)^12^. To determine whether any CYB5R family members contribute to ferroptosis, we performed knockdown (KD) experiments of CYB5R1, 2, 3, 4, and L in primary human aortic endothelial cells (HAECs) and then treated HAECs with RSL3, a ferroptosis inducer that inhibits and degrades glutathione peroxidase 4 (GPX4) (Extended Data Fig. 1a–b)^13,14^. Among the CYB5R family members, only CYB5R1 KD significantly increased susceptibility to RSL3 in HAECs (Extended Data Fig. 1b).

## CYB5R1 suppresses endothelial cell ferroptosis

**Extended Data Fig. 1:**
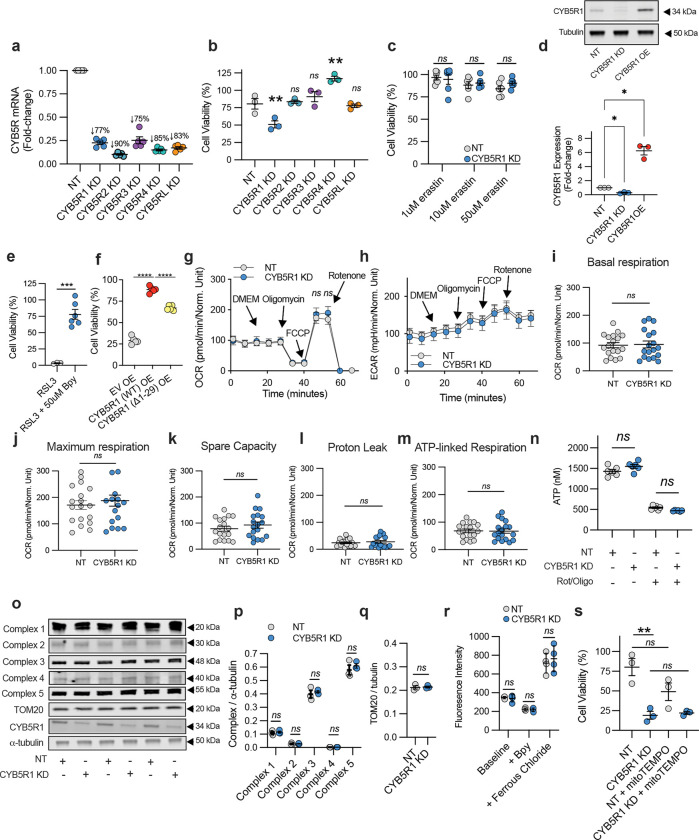
**a,** mRNA knockdown efficiency of each CYB5R isoform in HAECs. Data represent n = 5 technical replicates. **b,** RSL3-induced (500 nM) cell death in NT and CYB5R (1–5) KD HAECs. **c,** Dose-dependent toxicity of erastin in NT and CYB5R1 KD HAECs. **d,** Immunoblot analysis of CYB5R1 expression in NT, CYB5R1 KD, and CYB5R1 OE HAECs. Expression was normalized to α-tubulin. Quantification is the average of three independent experiments. **e**, RSL3-induced (1 μM) cell death in naive HAECs treated with 50 μM Bpy. **f**, RSL3-induced (500 nM) cell death in EV OE, CYB5R1 (WT) OE, CYB5R1 (Δ1–29) OE HAECs. **g-m**, Seahorse assay measuring mitochondrial respiration (**g**), glycolytic flux (**h**), basal respiration (**i**), maximum respiration (**j**), spare capacity (**k**), proton leak (**l**), and ATP-linked respiration (**m**) in NT and CYB5R1 KD HAECs. Data are mean ± s.e.m. of n = 18 technical replicates from one representative of two independently performed experiments. **n,** ATP luminescence assay to determine total (nM) ATP in NT and CYB5R1 KD HAECs. Rotenone/oligomycin (Rot/Oligo) were used as positive controls to deplete ATP. Data are mean ± s.e.m. of n = 6 technical replicates from one representative of one independently performed experiment. **o,** Immunoblot analysis of electron transport chain complex (1–5) and TOM20 expression in NT and CYB5R1 KD HAECs. Expression was normalized to α-tubulin. **o-p**, Quantification of electron transport chain complex (**p**) and TOM20 (**q**) protein expression from the immunoblot shown in **o**. Data are mean ± s.e.m. of n = 3 replicates from three separate cultures. **r**, FerroOrange assay to detect free ferrous iron in NT and CYB5R1 KD HAECs. Bpy, an iron chelator, was used as a negative control. Ferrous chloride was used as a positive control. Data are mean ± s.e.m. of n = 4 technical replicates from one representative experiment. **s,** RSL3-induced (500 nM) cell death with or without mitoTEMPO (5 μM) coincubation in NT and CYB5R1 KD HAECs. Cell viability data are mean ± s.e.m. of n = 3 (**b,s**), n= 5 (**e**), or n = 6 (**c,f**) wells of a 96-well plate from one representative of one independently performed experiment. ****P < 0.0001, ***P < 0.001, **P < 0.01, *P < 0.05, ns is not significant; students t-test (**f,i-m,q**), oneway ANOVA (**b,e,n),** RM one-way ANOVA (**d**), and two-way ANOVA (**c**,**g,h,p,r,s**).

To test whether CYB5R1 is necessary and sufficient to modulate ferroptosis, we performed knockdown and overexpression (OE) experiments in primary HAECs (Fig.1a, Extended Data Fig. 1d). CYB5R1 KD induced dose-dependent hypersensitivity to RSL3-induced ferroptosis, while CYB5R1 overexpression fully rescued HAECs from this RSL3-induced ferroptosis (Fig. 1b). Iron chelation with deferiprone (DFP) or 2,2′-bipyridyl (Bpy) and HAEC treatment with the lipid radical scavenger ferrostatin-1 (Fer-1) all prevented cell death, supporting a ferroptosis-driven mechanism (Fig. 1c, Extended Data Fig. 1e)^15^. However, these effects were erastinin-dependent, as in this system the Xc^−^ inhibitor^16^ failed to cause cell death (Extended Data Fig. 1c), thus pointing to the thiol-independence of this ferroptosis regulatory mechanism. Given that ferroptosis is driven by lipid oxidation, we assessed lipid hydroperoxide levels (LOOH) using Liperfluo^17^. CYB5R1 depletion significantly elevated RSL3-induced LOOH levels (Fig. 1d–f). Oxidized phosphatidylethanolamine species (PE) are key biomarkers of ferroptosis^17^. Consistent with this, LC-MS-based lipidomic profiling of CYB5R1 KD HAECs revealed elevated levels of multiple oxidized PE (PEox) species (Fig. 1g). This affirms that CYB5R1 is essential for regulating phospholipid oxidation and downstream ferroptosis in vascular endothelial cells.

Ferroptosis can be initiated in both cytosolic membrane compartments and mitochondria^18,19^. To define the subcellular localization of CYB5R1, the soybean peroxidase APEXII was fused to the *C*-terminus of CYB5R1. This enables the detection of focal hydroperoxide generation upon the oxidation of added 3,3′-diaminobenzidine (DAB)^20^. Wild-type (WT) CYB5R1 was enriched on the cytosolic side of the outer mitochondrial membrane (OMM) in HAECs (Fig. 1h–i). However, the truncated (Δ1–29) form of CYB5R1 lacks the membrane-targeting domain, thus disrupting mitochondrial localization and resulting in diffuse cytosolic distribution of CYB5R1 (Fig. 1i). Importantly, overexpression of Δ1–29 CYB5R1 did not confer full protection against RSL3-induced ferroptosis (Extended Data Fig. 1f), underscoring the necessity of a mitochondrial membrane localization for robust anti-ferroptotic functions of CYB5R1. Mitochondrial CYB5R1 localization was further defined by membrane fractionation (Fig. 1j) and fluorescence microscopy (Fig. 1k). The specific OMM localization of CYB5R1 prompted further investigation into its roles in mitochondrial function. Mitochondrial respiration (Extended Data Fig. 1g–l) and ATP production (Extended Data Fig. 1m–n) were comparable between non-targeted (NT) and CYB5R1 KD HAECs. Additionally, the expression of electron transport chain complex (I-V) proteins and TOM20, a mitochondrial marker were unchanged (Extended Data Fig. 1o–q). The labile ferrous iron pool was also unaffected by CYB5R1 KD (Extended Data Fig. 1r), indicating that changes in iron availability was not responsible for the increased sensitivity to ferroptosis. Scavenging mitochondrial ROS with MitoTEMPO also did not rescue RSL3-induced ferroptosis in CYB5R1 KD cells (Extended Data Fig. 1s), indicating that electron-transport-dependent partial reduction of oxygen to ROS does not induce ferroptosis in CYB5R1-deficient HAECs. Live-cell imaging revealed a distinct mitochondrial condensation and fragmentation phenotype in CYB5R1 KD HAECs upon RSL3 treatment (Fig. 1l–m), consistent with early morphological features of ferroptosis. These aggregate results indicate that CYB5R1 is a central regulator of ferroptosis in HAECs and that its localization to the OMM is essential for this function.

## CYB5R1 suppresses ferroptosis by reducing CoQ

**Extended Fig. 2:**
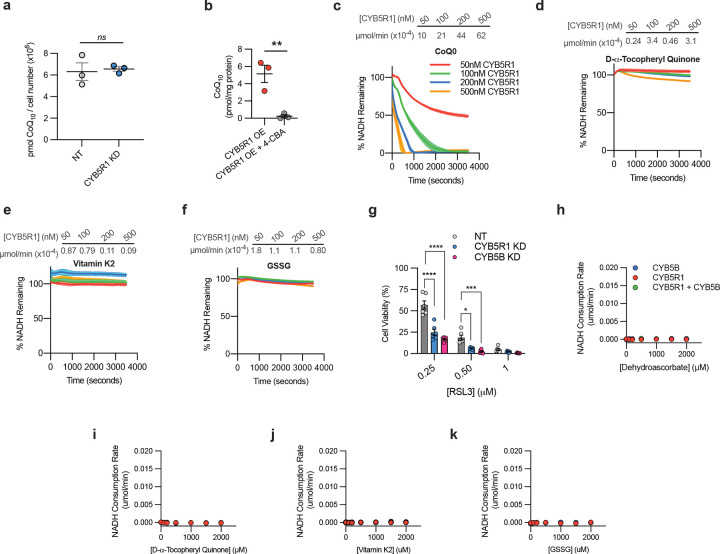
**a,** Quantification of total ubiquinol CoQ_10_ in NT and CYB5R1 KD HAECs using liquid chromatography–mass spectrometry. Ubiquinone 6 was used as an internal standard. **b**, Relative quantification of ubiquinol CoQ_10_ in CYB5R1 OE HAECs with or without 4-CBA (75 μM) treatment for 48 hrs. **c-f,** NADH consumption assay (340 nm) in tris buffer using varying concentrations of recombinant CYB5R1 (Δ1–29) in combination with various electron acceptor molecules - 2,3-dimethoxy-5-methyl-1,4-benzoquinone (CoQ_0_; **c**), D-α-Tocopheryl Quinone (oxidized Vitamin E; **d**), Vitamin K_2_ (**e**)_,_ and oxidized glutathione (GSSG; **f**). Rates of NADH consumption (μmol/min), at each CYB5R1 concentration, are shown above. Data are mean ± s.e.m. from the average of three independent experiments. **g**, Dose-dependent toxicity of RSL3-induced cell death of non-targeting (NT), CYB5R1 KD, and CYB5B KD HAECs. Data are mean ± s.e.m. of n = 6 wells of a 96-well plate from one independent experiment. **h-k,** NADH consumption assay with increasing substrate (dehydroascorbate (oxidized vitamin C; **h**), D-α-Tocopheryl Quinone (**i**), Vitamin K_2_ (**j**)_,_ and GSSG (**k**)) following the coincubation of recombinant CYB5R1 (Δ1–29) and CYB5B for 30 min at 37°C. Data are mean ± s.e.m. of n = 3 wells of one representative of one independently performed experiment. A non-linear regression model was used to fit the data, ****P < 0.0001, ***P < 0.001, **P < 0.01,*P < 0.05; student’s t-test (**b**) and two-way ANOVA (**a,g**).

Coenzyme Q_10_ (CoQ_10_, ubiquinone) is a lipophilic antioxidant. When fully reduced, CoQ_10_H_2_ (ubiquinol), is a radical scavenger and suppresses ferroptosis^21,22^. Its protective actions relies on the reduction of the fully oxidized and semi-quinone species ^23^. We hypothesized that CYB5R1 may suppress ferroptosis by promoting oxidized CoQ_10_ reduction. In RSL3-treated HAECs with CYB5R1 KD, the ratio of CoQ_10_H_2_/CoQ_10_ was significantly decreased (Fig. 2a), supporting a reliance on CYB5R1 in CoQ_10_ recycling. MS analysis of CYB5R1 KD HAECs showed no significant changes in total CoQ_10_ levels (Extended Data Fig. 2a), indicating that CYB5R1 does not impact CoQ_10_ biosynthesis. Pharmacological depletion of CoQ_10_ using 4-formylbenzoic acid (4-CBA), a CoQ2 biosynthetic enzyme inhibitor^24^, abolished the protective effect of CYB5R1 overexpression against ferroptosis (Fig. 2b–c and Extended Data Fig. 2b), thus confirming a dependence on CoQ_10_ for CYB5R1-mediated suppression of ferroptosis. In vitro, recombinant CYB5R1 readily reduced CoQ_0_, a water-soluble derivative of CoQ_10_ devoid of the isoprenoid side chain, in an NADH-dependent manner (Extended Data Fig. 2c). The reducing capacity of CYB5R1 is specific to CoQ, as other oxidized derivatives of common antioxidants (e.g., glutathione disulfide (GSSG), α-tocopheryl quinone and vitamin K_2_/menadione) were not reduced by CYB5R1 (Fig. 2d and Extended Data Fig. 2d–f). This underscores the significance of CoQ as a physiologically relevant substrate in the context of ferroptosis.

Cytochrome b5b (CYB5B), a hemoprotein located at the outer mitochondrial membrane, is an electron transfer partner with CYB5R3 and downstream acceptors^25^. It was evaluated whether CYB5R1 couples with CYB5B by genetic knockdown of CYB5B. This sensitized HAECs to RSL3-induced ferroptosis, similar to CYB5R1 KD (Extended Data Fig. 2g), suggesting that CYB5B is an electron transfer partner with CYB5R1 and CoQ. Recombinant CYB5B enhanced CoQ_0_ reduction by CYB5R1, increasing by 3x the V_max_ of NADH consumption (Fig. 2e). Other substrates tested were not reduced in the presence of CYB5B (Extended Data Fig. 2h–k). These results indicate that while CYB5B enhances CYB5R1-mediated CoQ_0_ reduction in vitro, it is not essential for this process.

Although CYB5R1 does not reduce α-tocopheryl quinone, it may reduce α-tocopherol radical with CoQ as the intermediate^26^. We therefore we tested whether CYB5R1 and CoQ_0_ could reduce Vitamin E radical (α-tocopherol radical) to α-tocopherol, a known inhibitor of ferroptosis. Using electron paramagnetic resonance (EPR) spectrometry, the combination of CYB5R1, CoQ_0_, and NADH suppressed Vitamin E radical formation (Fig. 2f–h) with NADH concentration dependency (Fig. 2h). The delayed onset of the EPR signal indicated progressive NADH consumption coupled with tocopherol radical reduction and the reduction of Vitamin E (Fig. 2h). This indicates that CYB5R1 reduces other radical species and catalyzes the NADH-dependent reduction of CoQ_10_ to suppress lipid oxidation and ferroptosis, with Vitamin E capable of accepting electrons from CoQ_10_H_2_.

Contradictory reports have proposed that CYB5R1 may promote ferroptosis by generating H_2_O_2_ through electron transfer to molecular oxygen^27^. Indeed, recombinant CYB5R1 generates H_2_O_2_; however, this effect was significantly diminished in the presence of CoQ_0_, which decreased H_2_O_2_ formation (Fig. 2i). This substrate-dependent activity was further revealed in HAECs treated with RSL3 and >9-fold CYB5R1 overexpression, where baseline phospholipid oxidation was elevated compared to empty vector (EV) overexpression cells (Fig. 2j). CoQ_10_ supplementation reversed phosphatidylethanolamine oxidation (Fig. 2j), underscoring the importance of CYB5R1-CoQ coupling in maintaining redox homeostasis. Together, these results reveal a redox bifurcation in which CYB5R1 acts as either an anti-ferroptotic or pro-ferroptotic enzyme, depending on the ratios of available electron acceptors including CoQ_10_ and CYB5B.

## CYB5R1 in atherosclerosis pathogenesis

**Extended Data Fig. 3:**
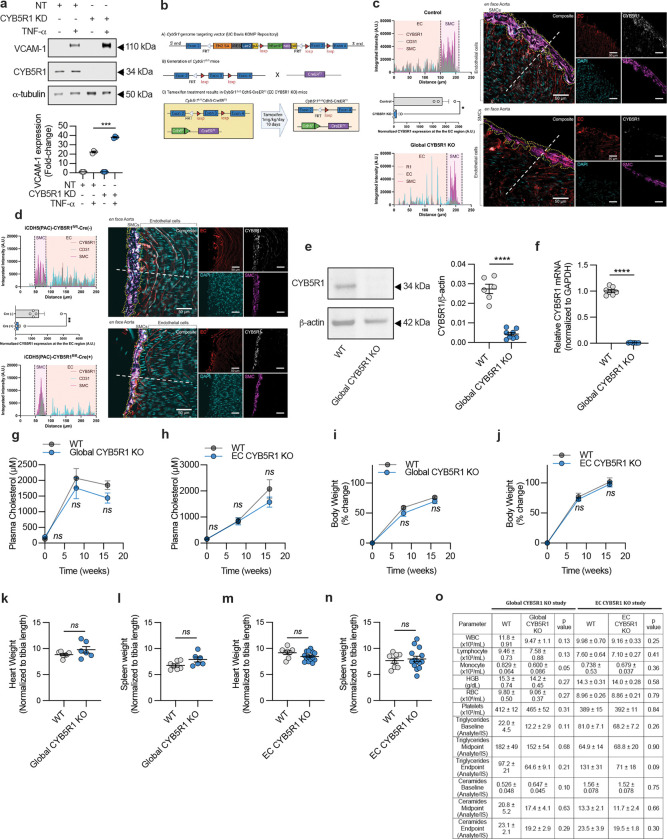
EC CYB5R1 KO mice develop severe atherosclerosis. **a**, Immunoblotting analysis of VCAM-1 in NT and CYB5R1 KD HAECs treated with TNF-α (10ng/mL for 18 hr). Protein expression was normalized to α-tubulin. Immunoblot images are cropped from the IR signal files. The immunoblot (top) and the quantification (bottom) is the one representative from three independently performed experiments. **b**, Generation of EC CYB5R1 KO mice. A) Schematic of genome targeting vector for CYB5R1 generated by the University of California (UC) Davis Knockout Mouse Repository (KOMP). Chimeras containing this targeting vector were crossed with FLPe recombinase mice to form CYB5R1^fl/fl^ mice. B) Schematic of CYB5R1^fl/fl^ mice which were crossed with Cdh5-CreER^T2^ mice. C) Tamoxifen treatment of CYB5R1^fl/fl^ Cdh5-CreER^T2^ mice results in excision of exon 3 of CYB5R1 solely in Cdh5 expressing cells resulting in EC-specific CYB5R1 knockout. **c-d**, Immunostaining analysis of global (**c**) and EC CYB5R1 (**d**) KO efficiency of *en face* aortas. Magenta: SMA-FITC, white: CYB5R1, red: PECAM, blue: DAPI. **e,** Immunoblotting analysis of aortas from WT (n=6) and global CYB5R1 KO (n=8) mice. Protein expression was normalized to actin. Immunoblot images are cropped from the IR signal files. Representative immunoblot is shown on the left. The quantification is shown on the right. **f**, Relative CYB5R1 mRNA levels in the heart from WT and global CYB5R1 KO mice. CYB5R1 mRNA was normalized to GAPDH. Quantification is the average from n=6–7 mice. **g-h**,Total plasma cholesterol content throughput the global (**g**) and EC CYB5R1 KO (**h**) atherosclerosis studies. **i-j**, Total body weights from the global (**i**) and EC CYB5R1 KO (**j**) atherosclerosis studies. **k-n**, Total heart and spleen weights of global (**k,l**) and EC CYB5R1 KO (**m,n**) mice at the endpoint of the atherosclerosis study. Total weights were normalized to tibia length. **o,** Heska measurements of circulating blood cells, triglycerides, and ceramides within plasma of mice at the endpoint of the global and EC CYB5R1 KO atherosclerosis studies, ****P < 0.0001, ***P < 0.001, *P < 0.05, ns is not significant; student’s t-test (**c,d**,**e,f,k,l,m,n,o**), one-way ANOVA (**a**), and two-way ANOVA (**g,h,i,j**).

Ferroptosis has been implicated in the early stages of inflammation and atherosclerosis^5,28^, contributing to early endothelial cell death. Consistent with this, genetic depletion of CYB5R1 in HAECs increased TNF-α-induced VCAM-1 expression (Extended Data Fig. 3a), suggesting that CYB5R1 plays a role in limiting pro-inflammatory signaling. Immunofluorescent staining showed markedly reduced expression of both CYB5R1 and GPX4 in human atherosclerotic coronary arteries (Fig. 3a–d, Extended Materials and Methods Table 1), pointing to a potential role for ferroptosis in plaque progression.

To further investigate the relationship between CYB5R1 and atherosclerosis, we generated global CYB5R1 and endothelial cell specific CYB5R1 knockout mice (Extended Data Fig. 3b–f). Mice were administered AAV8-D377Y-mPCSK9 and fed a 60% high-fat atherogenic diet to induce atherosclerosis (Fig. 3e). Oil Red O staining of aortic roots revealed a significant increase in plaque burden in both global and EC-specific CYB5R1 KO mice after 16 wk compared to wild-type controls (Fig. 3f–i), with no difference in total plasma cholesterol levels between the groups (Extended Data Fig. 3g–h). No significant differences in total body weight, heart and spleen weights, circulating blood cell counts, ceramides, and triglycerides between the groups were observed (Extended Data Fig. 3i–o). Collectively, these findings indicate that CYB5R1 plays a crucial role in suppressing atherogenesis.

## Quinone-nitroalkenes as ferroptosis inhibitors

The therapeutic potential of CoQ_10_ in suppressing ferroptosis is limited by its extreme hydrophobicity and poor bioavailability. Additionally, its efficacy may be further compromised in disease settings such as atherosclerosis, where reduced CYB5R1 expression can impair quinone redox cycling and exacerbate ferroptosis. To overcome these limitations, we designed and synthesized a bifunctional molecule with a shortened acyl chain that is a hybrid of a redox-active quinone with an electrophilic nitroalkene alkyl chain. This hybrid structure is intended to simultaneously mitigate oxidative stress via the quinone redox cycling and activate anti-inflammatory and cytoprotective pathways through electrophilic nitroalkene-mediated signaling via activation of protective signaling pathways including inhibition of NF-κB-dependent proinflammatory signaling and activation of Keap1/Nrf2-induced gene expression and the heat shock response^29,30^.

**Extended Data Fig. 4:**
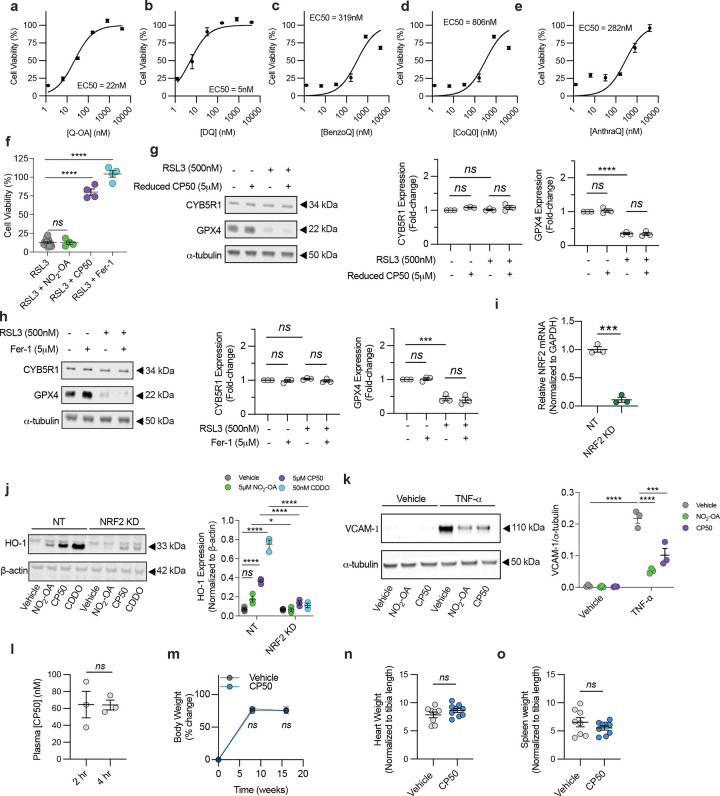
**a-e,** EC_50_ curves of Q-OA, DQ, BenzoQ, CoQ_0_, AnthraQ following cotreatment with RSL3 (500nM) in HAECs. To calculate EC_50_ values, curves were fit to a non-linear regression model comparing response to drug concentration. Data are mean ± s.e.m. of n = 5 wells of a 96-well plate from one representative of two independent experiments. To calculate EC_50_ values, curves were fit to a non-linear regression model comparing response to drug concentration. **f,** HAECs treated with RSL3 (500nM) in combination with 100 nM N_2_OA, CP50, or Fer-1. **g-h,** Immunoblotting analysis of HAECs treated with RSL3 in combination with reduced CP50 (**g**) or Fer-1 (**h**). CYB5R1 and GPX4 protein expression were normalized to α-tubulin. Immunoblot images are cropped from the IR signal files. The immunoblot (left) is one representative experiment; the quantification (right) is the average from three independent experiments. **i**, Relative mRNA levels of Nrf2 in NRF2 KD HAECs. **j**, Immunoblotting analysis of heme oxygenase-1 (HO-1) expression in NT and NRF2 KD HAECs treated with 5 μM NO_2_-OA, 5 μM CP50, or 50 nM CDDO. Immunoblot images are cropped from the IR signal files. The immunoblot (left) is one representative experiment. The quantification (right) is the average of n=3 technical replicates from one independently performed experiment. **k**, Immunoblotting analysis of vascular cell adhesion molecule-1 (VCAM-1) expression in HAECs treated TNF-α (10 ng/mL) with or without 5 μM NO_2_-OA or 5 μM CP50. Immunoblot images are cropped from the IR signal files. The immunoblot (left) is one represntative experiment. The quantification (right) is the average of n=3 technical replicates from one independently performed experiment. **l**, Plasma [CP50] 2 and 4 hr post oral gavage (50 mg/kg) determined by LC-MS. **m**, Total body weights from the CP50 atherosclerosis study. **n-o**,Total heart (**n**) and spleen weights (**o**) of vehicle and CP50 mice at the endpoint of the atherosclerosis study, ****P < 0.0001, ***P < 0.001, *P < 0.05, ns is not significant; one-way ANOVA (**f**), two-way ANOVA (**g,h,j,k,m**) or student’s t-test (**i,l,n,o**).

To dissect the individual and combined contributions of these two reactive motifs, we synthesized and screened a panel of analogs: decyl-ubiquinone (DQ) and a quinone–alkene (Q–OA), which retain quinone-dependent activity but lack the electrophilic nitroalkene moiety, and nitro-oleic acid (NO_2_-OA), which contains the electrophilic functional group but lacks the quinone core. Among these, only the compounds containing a quinone head, such as quinone–nitroalkene hybrid (CP50) and, DQ and Q–OA, conferred potent protection against RSL3-induced ferroptosis in HAECs, with CP50 displaying a low nM EC₅₀ comparable to ferrostatin-1 (Fig. 4a–c, Extended Data Fig. 4a–b). In contrast, CoQ₀, lacking the acyl chain, exerts no protection, likely due to limited mitochondrial membrane association. Additionally, benzoquinone–nitroalkene (BenzoQ) and anthraquinone–nitroalkene (AnthraQ) were not protective against ferroptosis indicating that benzoquinone and anthraquinone moieties were less redox reactive under these conditions (Fig. 4a, Extended Data Fig. 4c–e). Removal of the CP50 quinone moiety (giving NO_2_-OA) did not rescue RSL3-induced ferroptosis (Extended Data Fig. 4f), with CP50 mitigating liperfluo oxidation in HAECs (Fig. 4d). These data indicate that CP50 suppresses ferroptosis through quinone redox cycling.

While the quinone functional group of CP50 exerts the anti-ferroptotic effect, the nitroalkene moiety upregulated CYB5R1 protein expression and prevented GPX4 degradation (Fig. 4e–f), effects not observed with the non-electrophilic analog, Q-OA (Extended Data Fig. 4g) or ferrostatin-1 (Extended Data Fig. 4h). Furthermore, CP50 activated Nrf2-regulated gene expression, as evidenced by increased heme oxygenase-1 expression in control but not Nrf2 knockdown HAECs (Extended Data Fig. 4i–j). CP50 also inhibited TNF-α–induced VCAM-1 expression, indicating suppression of NF-κB–mediated inflammation (Extended Data Fig. 4k). These findings demonstrate that the quinone moiety of CP50 is indispensable for ferroptosis suppression, while its nitroalkene group is critical for activating electrophile-responsive adaptive cytoprotective pathways. This dual functionality positions CP50 as a promising therapeutic candidate for atherosclerosis and other inflammatory-related disorders.

The anti-atherosclerotic effect of CP50 was tested in mice injected with AAV8-D377Y-mPCSK9 and fed a 60% high-fat diet for 16 wk (Fig. 4g). Beginning at wk 12, mice received oral CP50 (50 mg/kg) every other day for 4 wk, achieving plasma concentrations of ~60 nM at 4 hr post-dosing (Extended Data Fig. 4l). CP50 treatment significantly reduced aortic plaque area, as assessed by Oil Red O staining (Fig. 4h–i), without affecting plasma cholesterol, body weight, heart weight, or spleen weight (Extended Data Fig. 4m–p). These findings suggest that CP50 may hold therapeutic promise for limiting plaque progression in atherosclerosis.

In summary, these findings reveal that CYB5R1 establishes a redox relay with CoQ_10_ at the outer mitochondrial membrane to protect endothelial cells from ferroptosis and atherosclerosis (Fig. 4j). This defense mechanism may extend beyond the vasculature, modulating ferroptosis susceptibility across diverse cell types and disease contexts. Moreover, we reveal CYB5R1 to be either an anti- or pro-ferroptotic enzyme depending on electron acceptor context, a principle that likely extends to other CoQ reductases, including FSP-1 and NQO1, that may operate through analogous redox mechanisms. We also identify a novel quinone–nitroalkene hybrid with potent anti-ferroptotic and anti-atherosclerotic activity. Finally, genotype–phenotype screening for CYB5R1 loss-of-function variants may offer predictive insights into individual susceptibility to ferroptosis-driven diseases, positioning CYB5R1 as a promising therapeutic target across a spectrum of ferroptosis-associated pathologies.

## Methods Summary

Primary human aortic endothelial cells were maintained at low passage and subjected to CYB5R1 loss- and gain-of-function approaches using siRNA knockdown or CMV-driven overexpression, including soluble and APEX2-tagged variants^31^. Recombinant soluble CYB5R1 was expressed in E. coli and purified for biochemical assays^32^. A quinone–nitroalkene compound (CP50) and a reduced analog were synthesized and validated by NMR and mass spectrometry. Cell viability under ferroptotic stressors was monitored by WST-8 assay, with Coenzyme Q (CoQ) depletion or rescue achieved through pharmacological inhibition or CoQ-liposome supplementation. Protein expression and localization were assessed by immunoblotting, organelle fractionation, confocal imaging, and APEX2-based electron microscopy^33^. Lipid peroxidation was detected by fluorescent probes^17^, and mitochondrial morphology tracked by live-cell microscopy. Targeted lipidomics, redox lipidomics, and CoQ redox states were quantified by LC-MS/MS with internal standards^34,35^. Enzymatic assays measured CYB5R1-dependent NADH oxidation and hydrogen peroxide formation. Cellular bioenergetics were analyzed by Seahorse XF^36^, with parallel ATP, cell number, and labile iron quantification. Radical intermediates were studied by EPR in a lipid oxidation model^37,38^. For in vivo studies, endothelial-specific and global *Cyb5r1* knockout mice were generated and subjected to PCSK9-induced, diet-driven atherosclerosis^39–41^. Lesion burden was quantified by Oil Red O staining, with plasma lipids, ceramides, and hematology profiled by LCMS/MS and automated analyzers. Human coronary artery tissues were stained for CYB5R1 expression. Statistical analyses used standard tests with significance at p < 0.05. Detailed methods can be found in extended materials and methods.

## Extended Materials and Methods

### Cell lines.

All cells were cultured in a 37°C humidified incubator under 5% CO_2_. Human aortic endothelial cells (HAEC) were purchased commercially (Lonza, CC-2535) and cultured in EBM-2 cell growth basal media supplemented with EGM^®^−2 Endothelial SingleQuots^®^ Kit (Lonza, CC-3162). Cells were not used experimentally above a population doubling of 13.

### Cloning of expression vectors.

Empty overexpression vector pCIG3 was obtained commercially (Addgene, 78264). The original clone generated from pCIG3 showed low promoter activity, therefore using standard subcloning protocols we substituted the original CMV promoter with the CMV promoter region containing an enhancer from pcDNA3.1 (Invitrogen, V86020). The modified plasmid was confirmed with whole plasmid sequencing (Genewiz, Plasmid-EZ) and named pCIGX. The wild-type (WT) human *Cyb5r1* (NM_016243.3) was subcloned into the pCIGX vector. An APEXII containing plasmid was purchased (Addgene, 85824) and the *cyb5r1* coding sequence with a GGGGS amino acid linker repeated 3 times (VectorBuilder, VB211104–1299nub) was subcloned upstream of the C-terminal APEXII tag to create CYB5R1-APEXII constructs. The soluble version of *Cyb5r1* (Δ1–29) was created by deleting the first 29 amino acids containing the membrane anchor via PCR prior to insertion into pCIGX, pET His 6 SUMO (Addgene 29659) and the APEXII plasmids. All construct sequences were verified by Sanger sequencing (Genewiz).

### Generation of recombinant CYB5R1 protein.

The pET His 6 SUMO-Δ1–29 CYB5R1 plasmid was transformed into SoluBL21^™^ Competent E. coli competent cells (Amsbio, AMS.C700200) and cultured in LB broth overnight at 37°C. Starter cultures were diluted 1:100 in LB and grown at 37°C until reaching an OD_600_ of 0.6–1.0. Expression was induced with 1 mM IPTG, and protein was expressed at 23°C for 16 hr. Cells were harvested by centrifugation and lysed by sonication in 50 mM Tri-HCl, 150 mM NaCl, 10% v/v glycerol, pH 7.4 on ice. Soluble protein was separated by centrifugation at 25,000 × g, 4°C for 30 min and the supernatant was applied to a NiNTA gravity column equilibrated with lysis buffer. The column was washed in lysis buffer supplemented with 40 mM imidazole and the protein was eluted in lysis buffer supplemented with 400 mM imidazole. Fractions containing the CYB5R1 protein were combined and concentrated using Amicon Ultra 30k centrifugal concentrators (Millipore, UFC9030). The His-tagged Smt3-specific protease, Ulp1, was added to cleave the fusion and the mixture was applied to a Sephacryl S-300 HR size exclusion column (Cytiva, 1711960) equilibrated in lysis buffer. The protein mixture was reapplied to a NiNTA column to capture the His-tagged Smt3 and Ulp1 proteins. Flow-through containing the CYB5R1 protein was collected, and a final buffer exchange was performed using a Superdex 75 Increase 10/300 GL (Cytiva, 29148721). Protein was concentrated, flash-frozen in liquid N2 and stored at −80°C prior to use.

### Transfection of siRNA and expression vectors.

The transfection of siRNA and expression vectors in HAECs were achieved using Lipofectamine 3000 (Thermo Fisher Scientific, L3000015) according to the manufacturer’s instructions. Non-targeting Silencer Select siRNA (siNT) and human *Cyb5r1* targeting siRNA (siCYB5R1) were purchased from Thermo Fisher (4390843 and 4392420 s224233). Overexpression vectors were cloned as described above. HAECs were seeded at 15,000 cells/cm^2^ 24 hr prior to transfection. For knockdown, HAECs were treated with siRNA:lipofectamine 3000 complex overnight, before the medium was replenished and cells cultured for 72 hr to achieve maximal knockdown efficiency. For overexpression studies, HAECs were pretreated 30 min with 5 uM MRT67307, a STING pathway inhibitor that promotes the uptake of exogenous DNA^42^. Lipofectamine complexes were prepared using 0.2 ug plasmid DNA per well of a 6 well plate and applied to cells for 4 hours. Transfection mixes were then removed and replaced with medium containing 5 uM MRT67307. The following day, fresh medium lacking MRT67307 was added to the cells and allowed to incubate for an additional 24 hr. For excess (>9-fold) overexpression studies, lipofectamine complexes were prepared using the MRT67307 method and 0.4 ug plasmid DNA per well of a 6 well plate and applied to the cells for 4 hours.

### Synthesis of CP50 and non-electrophilic CP50.

**Scheme 1:**
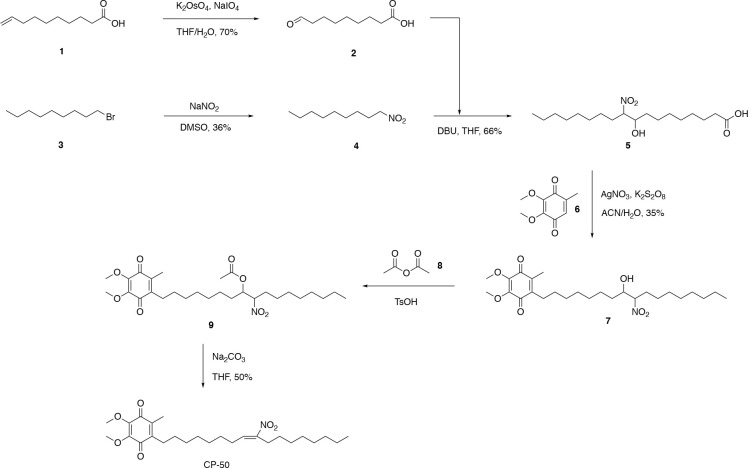


The synthesis of CP50 was supported by ACME Bioscience, Inc. (A Frontage Lab company). The synthetic route was shown in Scheme 1.

#### Synthetic Procedures

##### Synthesis of Compound **2**

In a 1000-mL flask, compound **1** (50.0 g, 294 mmol, 1.00 eq) was dissolved in tetrahydrofuran (1500 mL) and water (750 mL). The solution was cooled down in an ice-water bath before a solution of potassium osmate (1.62 g, 4.41 mmol, 0.0150 eq) in water (32 mL) was added to the reaction solution. Sodium periodate (160 g, 735 mmol, 2.50 eq) was added in three portions every 30 min and the reaction was kept in ice-water bath for 3 h before it warmed up to room temperature overnight. The reaction solution was filtered, and the solid precipitate was washed with ethyl acetate (1.2 L × 3). The water phase was separated and mixed with saturated sodium thiosulfate solution (320 mL). To the aqueous solution was added conc. hydrochloric acid (20 mL) to adjust the solution to pH 5. It was extracted with ethyl acetate (300 mL × 2). The combined organic phases were washed with brine solution (200 mL × 2), dried over anhydrous sodium sulfate, and concentrated under reduced pressure. The residue was purified by SiO_2_ column chromatography to give compound **2** (38 g, 70%).

##### Synthesis of Compound **4**

Sodium nitrite (50 g, 0.73 mol, 1.5 eq) was added in dimethyl sulfoxide (500 mL) in a 1000-mL flask. A homogenous solution was obtained in 4 h before compound **3** (100 g, 0.480 mol, 1.00 eq) was slowly added. The mixture was stirred at rt for 2 h. The solution was diluted with water (2 L) and extracted with ethyl acetate (500 mL × 2). The combined organic phases were washed with brine solution (300 mL × 2), dried over sodium sulfate, and concentrated to give a residue, which was purified by SiO_2_ column chromatography (2–8% ethyl acetate in hexanes) to give compound **4** (45 g, 36%).

##### Synthesis of Compound **5**

In a 250-mL flask, compound **2** (34.0 g, 198 mmol, 1.00 eq), compound **4** (35.9 g, 208 mmol, 1.05 eq), and 1,8-diazabicyclo[5.4.0]undec-7-ene (24.1 g, 158 mmol, 0.800 eq) were mixed in tetrahydrofuran (100 mL). The reaction flask was kept at rt with a water bath for 30 min. The reaction was stirred overnight. NMR indicated the reaction was completed. The mixture was dissolved in saturated NH_4_Cl aqueous solution and concentrated HCl (5 mL) mixture and extracted with CH_2_Cl_2_ (200 mL × 3). The combined organic phases were washed with brine before being dried over magnesium sulfate, filtered, and evaporated to dryness. The crude product was purified with SiO_2_ column chromatography to give compound **5** (44.5 g, 66%).

##### Synthesis of Compound **7**

To a solution of compound **6** (23.7 g, 130 mmol, 1.50 eq) in ACN (300 mL) at rt was added compound **5** (30.0 g, 86.8 mmol, 1.00 eq) and silver nitrate (5.9 g, 34.7 mmol, 0.4 eq). The mixture was heated to 90 °C. Potassium persulfate (47.0 g in water, 174 mmol) was added dropwise. The mixture was stirred at 90 °C for 2 days. The mixture was diluted with ethyl acetate/water (600 mL/300 mL) and the water phase was extracted with ethyl acetate (200 mL × 2). The combined organics were washed with water (300 mL) and brine (300 mL), dried over sodium sulfate, and concentrated under vacuum. The crude was purified by SiO_2_ column chromatography (5–10% ethyl acetate in hexanes) to give compound **7** (14.1 g, 35%).

##### Synthesis of Compound **9**

To a solution of compound **7** (20.0 g, 41.6 mmol, 1.00 eq) in acetic anhydride (200 mL) was added *p*-toluenesulfonic acid monohydrate (3.58 g, 20.8 mmol, 0.500 eq). The solution was stirred at rt for 4 h. After removal of acetic anhydride, diethyl ether (300 mL) was added. The organic layer was washed with water (200 mL) and brine (200 mL), dried over sodium sulfate, and concentrated to give crude compound **9** (20.2 g), which was used for the next step without further purification.

##### Synthesis of CP50

To a solution of compound **9** (20.0 g, 38.6 mmol, 1.00 eq) in tetrahydrofuran (200 mL) was added sodium carbonate (4.91 g, 46.3 mmol, 1.20 eq). The mixture was stirred at 70 °C for 48 h. After being cooled to rt, the mixture was diluted with ethyl acetate/water (500 mL/500 mL). The aqueous phase was extracted with ethyl acetate (100 mL × 2). The combined organics were washed with water (200 mL) and brine (200 mL), dried over sodium sulfate, and concentrated. The crude was purified with SiO_2_ column chromatography (2–5% ethyl acetate in hexanes) to give CP-50 (9.67 g, 50% over two steps) as an orange oil. [M+H]^+^ 464.45, [M+Na]^+^ 486.30. ^1^H NMR (400 MHz, CDCl_3_) 7.06–7.02 (m, 1H), 3.97 (s, 6H), 2.56–2.52 (m, 2H), 2.44–2.41 (m, 2H), 2.20–2.16 (m, 2H) 1.99 (s, 3H), 1.49–1.41 (m, 4H), 1.34–1.32 (m, 8H), 1.26–1.24 (m, 10H), 0.87–0.84 (m, 3H).

##### Synthesis of non-electrophilic reduced CP50


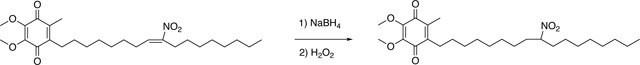


To a solution of CP50 (23 mg, 0.049 mmol) in THF (3 mL) at 0 °C was added NaBH_4_ (11 mg, 0.29 mmol). The reaction was stirred at rt for 2 h before being quenched with a saturated NH_4_Cl aq. solution. The mixture was extracted with dichloromethane three times. The combined organic layers were dried and evaporated. In case of the auto-oxidation of benzoquinone during the reaction workup was incomplete, the crude product was dissolved in dichloromethane (3 mL) and 3wt% H_2_O_2_ solution (3 mL) and stirred at rt overnight. The resulting mixture was then phase separated. The organic layer was washed twice with H_2_O. The organic layer was dried with Na_2_SO_4_, filtered, and evaporated. The crude product was purified by a SiO_2_ column (0 – 15% ethyl acetate in hexanes) following 280 nm absorption, giving the desired product as a yellow oil (15 mg, 65%). ^1^H NMR (300 MHz, CDCl_3_) d 0.87 (t, J = 6.9Hz, 3H), 1.19–1.42 (m, 24H), 1.68 (m, 2H), 1.93 (m, 2H). 2.01 (s, 3H), 2.44 (t, J = 7.0Hz, 2H), 3.98 (s, 3H), 3.99 (s, 3H), 4.45 (m, 1H); ^13^C NMR (75 MHz, CDCl3) d 11.9, 14.1, 22.6, 25.7, 25.8, 26.4, 28.7, 28.9, 29.0, 29.1, 29.1, 29.1, 29.2, 29.7, 31.8, 33.9, 33.9, 61.1, 89.1, 138.7, 143.0, 144.3, 184.2, 184.7.

**Figure 1.**
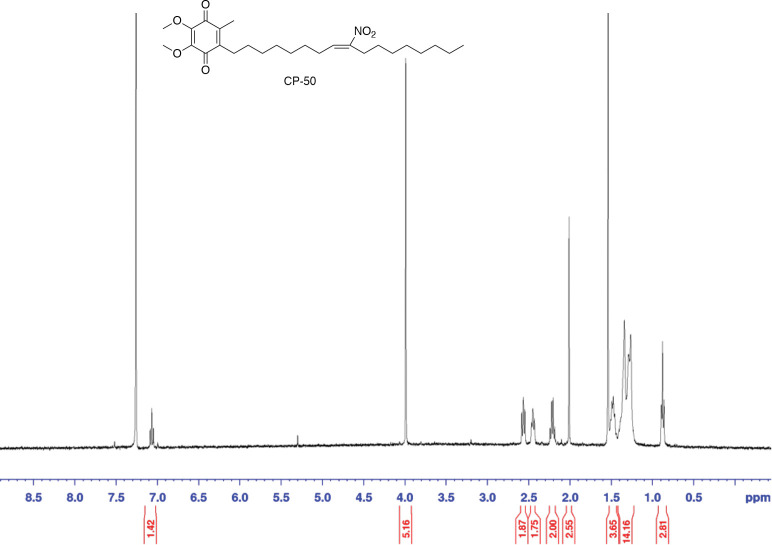
^1^HNMR of CP50 in CDCl_3_

**Figure 2.**
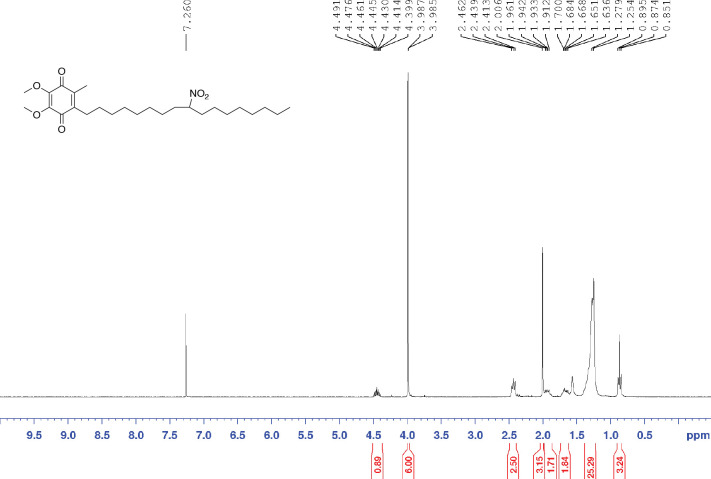
^1^HNMR of reduced CP50 in CDCl_3_.

**Figure 3.**
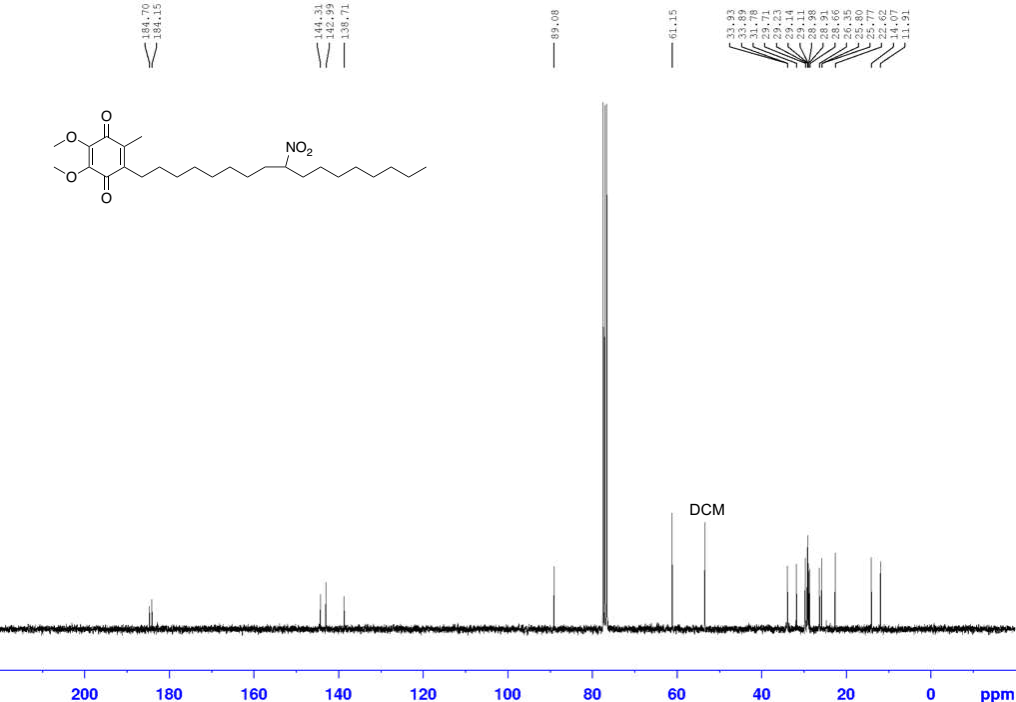
^13^CNMR of reduced CP50 in CDCl_3_.

### Cell viability assays.

HAECs were seeded on 96-well plated at 10,000 cells/cm^2^ to achieve 50% confluence the following day. Cells were then treated with varying concentrations of test compounds (RSL3, erastin, deferiprone (DFP), ferrostatin-1 (Fer-I), CP50, Q-OA, CoQ_0_, DQ, BenzoQ, AnthraQ) for 20 hours in media lacking ascorbic acid. Cell viability was assessed by adding 0.5 mM WST-8/ 20 uM PMS, incubating 4 hours and measuring the Absorbance at 450 nm (A_450_). A_450_ of untreated cells was used for 100% viability. For experiments including mitoTEMPO, 5 μM mitoTEMPO was incubated with the cells for 1 hr at 37°C prior to adding RSL3.

### Depletion of Coenzyme Q *in vitro*.

Depletion of CoQ_10_ in HAECs was achieved through the treatment with 4-formylbenzoic acid (4-CBA), an inhibitor of the CoQ2 enzyme that is responsible for the rate limiting step in CoQ biosynthesis. CYB5R1 overexpressing cells were plated at 10,000 cells/cm^2^ and treated with vehicle (1% v/v DMSO) or 75 μM 4-CBA for 24 hours. The next day, medium was replaced with fresh medium lacking ascorbic acid supplemented with 4-CBA and RSL3. Cell viability was assessed 24 hr after treatment using the WST-8/PMS assay. Cells were cultured in 10 μM uridine to complement the loss of CoQ.

### Immunoblotting.

HAECs were lysed directly in 2x Laemmli buffer and boiled at 95°C for 10 min. Equal volumes of lysate were loaded into NuPAGE 4%–12% Bis-Tris Gels (Thermo Fisher, NP0335BOX) and underwent electrophoresis at 180V for 75 minutes completed with MOPS Running Buffer (Thermo Fisher, NP0001). Proteins were transferred to 0.2 um nitrocellulose membranes in 25 mM Tris-192mM Glycine buffer for 1.5 hours at 100V. Membranes were blocked in LI-COR Intercept PBS Blocking Buffer (LI-COR, 927–40003) diluted 1:1 with PBS (pH 7.4), followed by blotting overnight at 4°C with primary antibody in 1:1 LI-COR Intercept PBS Blocking Buffer/PBST (0.1% Tween PBS pH 7.4). Membranes were washed and incubated in LI-COR IR dye conjugated secondary antibodies for 1 hour at room temperature, followed by several washes with PBST and signal detection with a LI-COR Odyssey imaging system. Integrated intensities were quantified using Image Studio Lite (LI-COR), and protein expression was normalized to a house keeping gene.

### Membrane fractionation assay.

HAECs were seeded in a 10 cm dish and cultured to confluency. HAECs were trypsinized and subjected to organelle fractionation according to the manufacturer’s instructions (Qiagen, 37612). Once isolated, immunoblotting was performed for CYB5R1 along with tubulin and tom20 as positive controls for the cytosol and mitochondria, respectively.

### Immunostaining.

For cells, transient expression of APEXII tagged CYB5R1 was achieved by transfecting HAECs with the pCIGX-CYB5R1-APEXII plasmid with Lipofectamine 3000. The next day, transfected HAECs were re-plated onto poly-l-lysine coated glass coverslips and allowed to adhere overnight at 37°C/5% CO_2_. The following day, cells were incubated with 250 nM MitoTracker Deep Red fluorescent dye (Thermo Fisher, M22426). Cells were washed 3x with PBS, fixed for 15 min in PBS containing 4% (w/v) paraformaldehyde, and washed again 3x with PBS. Cells were permeabilized for 20 min with PBS containing 0.10% triton X-100 and washed 3x with PBS. Cells were incubated in PBS blocking solution (1% BSA/0.10% Tween) for 45 min at room temperature in the dark followed by an incubation in APEX2 primary antibody (1:100) diluted in PBS with 1% BSA/0.10% tween for 1 hr at room temperature in the dark. Cells were washed 3x with PBS and incubated with AlexaFluor594 (1:1000) diluted in PBS (1% BSA, 0.10% tween) for 1 hr at room temperature in the dark. Finally, cells were washed 3x with PBS prior to ImmunoFluorescent imaging. Image files were rendered to 3D using IMARIS software.

For studies using mouse tissue, aortas were collected from experimental animals and fixed in 100% methanol for *en face* immunostaining. Tissues were permeabilized with 0.01% Tween-20 in PBS and blocked with 10% horse serum, 1% BSA, and 0.01% Tween-20 in PBS. Samples were stained with DAPI for nuclei, FITC-conjugated smooth muscle α-actin for smooth muscle, goat anti-PECAM1 for endothelium, and rabbit anti-CYB5R1 followed by Alexa Fluor 647-conjugated donkey anti-rabbit secondary antibody. Goat anti-PECAM1 was detected with Alexa Fluor 594-conjugated donkey anti-goat secondary antibody. Confocal images were acquired using a Leica Stellaris 5 microscope (Leica Microsystems, Buffalo Grove, IL), post-processed with Leica LAS X software, and analyzed using Fiji (ImageJ). Z-stack images spanning the endothelial cell (EC) layer were collected (6.58 μm total thickness, 20 z-steps) using a Leica HC PL APO 40x/1.30 Oil objective. EC and smooth muscle cell (SMC) masks were generated from PECAM1 and SMAA channels using Otsu thresholding. A difference mask was applied to eliminate overlap between EC and SMC regions, allowing quantification of the CYB5R1 signal specifically within the EC region. To visualize spatial signal distribution, integrated line profiles of PECAM1, SMAA, and CYB5R1 fluorescence were generated from SUM projection images.

For the human atherosclerosis experiments, human coronary arteries were embedded in paraffin and cut to a thickness of 10 μm. Deparaffinization was achieved by warming the tissues to 65°C, followed by baths in xylene and graded alcohol for paraffin extraction. Deparaffinized tissues were rehydrated in distilled H2O and boiled for 20 minutes in antigen-unmasking solution (Vector Labs, H-3300). The cooled samples were then washed with PBS and blocked with PBS containing 3% fish skin gelatin and 10% horse serum (Sigma, H1270) for one hour. Primary antibodies were diluted in the blocking solution and incubated overnight at 4°C. The primary antibodies were washed three times with PBS containing 3% fish skin gelatin (Sigma-Aldrich, G7765) and 0.1% TWEEN 20 (Fisher Scientific, BP337 100). The secondary antibody Fluor 594 Goat anti-rabbit (ThermoFisher, A-11012) was diluted in a blocking solution, incubated for one hour protected from light, and then washed three times for five minutes each. Normal rabbit polyclonal IgG (Invitrogen, 31235) was used as a negative isotype antibody control at the same concentration as the specific antibodies. Specimens were finally mounted with Fluoroshield Mounting Medium with DAPI (Abcam) and imaged within 24 hours. Images were obtained using an EVOS FL microscope (Life Technologies, AMA3300) at 10x magnification with an EVOS Plan Fluor 10x/0.5 objective (Life Technologies, AMEP 4698) at 360/447 nm excitation/emission (DAPI) and 530/593 nm excitation/emission (RFP) filters. Fluorescence intensity quantification was performed using ImageJ version 1.54p and normalized to the DAPI signal. Representative images were selected based on quality, and the average of the quantification across multiple images was calculated.

### Assessment of lipid peroxidation using Liperfluo.

The day before imaging, HAECs were seeded on 24 well plates and grown to 50% confluency. Cells were washed once with serum free medium and incubated in ascorbic acid-free HAEC medium supplemented with 500 nM RSL3 or DMSO vehicle for 2 hr. In some studies, 1 μM CP50 was added simultaneously with RSL3. Treatments were removed, cells were washed 2x with 1x HBSS, and incubated with 5 μM Liperfluo (Dojindo, L248) for 30 min at 37°C/5% CO_2_. The solution was removed and cells were washed 3x with 1x HBSS prior to imaging. Fluorescent microscopy was performed using a LEICA epifluorescent microscope. Fiji was used to quantify 525 nm fluorescence values for each cell and corrected for background by subtracting the green fluorescence in cell-free areas. The Liperfluo intensity values were calculated and normalized to cell area. Brightfield images were used to draw outlines around each individual cell using Cellpose.

### Live-cell confocal microscopy to investigate mitochondrial morphology.

NT and CYB5R1 KD HAECs were seeded on poly-l-lysine glass coverslips in 6 well dishes and grown to 50% confluency overnight at 37°C/5% CO_2_. The next day, cells were washed 3x with PBS and loaded with 1 mL HBSS prior to imaging. After the first frame was recorded (6 sec), 1 μM RSL3 was added to the cells and live images were captured every 2 min. Quantification of mitochondrial perimeter and form factor at each time point were performed in Fiji.

### Phospholipidomics and redox phospholipidomics LC-MS/MS analysis in cells.

Lipids from HAEC cells were extracted using the Folch procedure [1] and phosphorus [2] was determined by a micro-method. Briefly, 0.5 × 10^6^ cells were resuspended in 0.75% KCl and lipids were extracted using a 2:1 v/v chloroform:methanol mixture. To prevent oxidation of lipids during extraction and sample preparation for LC/MS analysis, a chloroform-methanol mixture containing 0.01% butylated hydroxytoluene (BHT) was used. LC/ESI-MS analyses of lipids, oxygenated lipids as well as oxidatively truncated species were performed on a Thermo HPLC system coupled to either an Orbitrap Fusion Lumos mass spectrometer (Thermo Fisher Scientific) for normal phase analysis or a QExactive mass spectrometer (Thermo Fisher Scientific) for reverse-phase (C30) analysis. Normal phase analysis was performed on a silica column (Luna 3 μm Silica (2) 100 A, 150 × 1.0 mm, (Phenomenex)) at a flow rate of 0.065 ml/min. The column was maintained at 35°C. For normal phase analysis, lipids were analyzed in negative ion mode using the following parameters: capillary voltage, 3500; sheath, aux and sweep gases (35, 17, 0, 9 respectively); ion transfer tube temperature, 300°C; orbitrap resolution, 120,000; scan range 400–1800 m/z. For data dependent MS2, an isolation window of 1.2 m/z was used. Collision energy (HCD) was static at 24 with an orbitrap resolution of 15,000. To assess isomeric species, lipids and oxygenated lipids were separated on a C30 reverse phase column (Accucore 2.6 μm, 2.1mm × 25 cm, Thermo Scientific) at a flow rate of 0.1 mL/min. The column was eluted using a gradient solvent system consisting of mobile phase A: acetonitrile/water (50/50); mobile phase B: 2-propanol/acetonitile/water (85/10/5). Both A and B solvents contained 5mM ammonium formate and 0.1% formic acid as modifiers. Gradient method was as follows: 0–40 min, 15%–50% B (linear, 5); 40–130 min, 50–100% B (linear, 5); 130–135 min, hold at 100% B; 135–140 min, 15% B (linear, 5); 140–150 min, 15% B for equilibration. Column temperature was set at 35°C. For C30 analysis, lipids were analyzed in negative ion mode on a Q-Exactive mass spectrometer using the following parameters; capillary voltage, 3500; sheath, aux and sweep gases (12, 0, 0, respectively); ion transfer tube temperature, 320°C; orbitrap resolution, 140,000; scan range 150–1800 m/z. Collision energy (HCD) was static at 24 with an orbitrap resolution of 17,500. Analysis of LC/MS data was performed using Compound DiscovererTM 2.0 software package (Thermo Fisher Scientific, San Jose, CA) with an in-house generated analysis workflow and oxidized phospholipid database. Peaks with signal/noise ratio of more than 3 were identified and searched against non-oxidized and oxidized phospholipid database. The species assignments were done based on three criteria: retention time, exact mass, and fragmentation analysis. Values for m/z were matched within 5 ppm to identify the lipid species. The structure of identified lipids was confirmed by fragmentation analysis, unless otherwise specified. The retention time of each lipid class for normal phase analysis was determined based on the retention time of the exogenously added internal standard. Deuterated phospholipids: 1-hexadecanoyl(d_31_)-2-(9Z-octadecenoyl)-snglycero-3-phospho-ethanolamine (PE(16:0D_31_/18:1)), 1-hexadecanoyl(d_31_)-2-(9Z-octadecenoyl)-sn-glycero-3-phosphocholine (PC(16:0D_31_/18:1)), 1-hexadecanoyl(d_31_)-2-(9Z-octadecenoyl)-sn-glycero-3-phosphoserine (PS(16:0D_31_/18:1)), 1-hexa-decanoyl(d_31_)-2-(9Z-octadecenoyl)-snglycero-3-phosphate (PA(16:0D_31_/18:1)), 1-hexadecanoyl(d_31_)-2-(9Zoctadecenoyl)-sn-glycero-3-phosphoglycerol (PG(16:0D31/18:1)), 1-hexadecanoyl(d_31_)-2-(9Z-octadecenoyl)-snglycero-3-phospho-(1′-myo-inositol) (PI(16:0D_31_/18:1)) and 1,1′,2,2′-tetramyristoylcardiolipin (sodium salt) from Avanti Polar Lipids (Alabaster, AL) were used as internal standards. Internal standards were added directly to the MS sample to a final concentration of 1 μM. Peak areas were used for quantification of phospholipids and oxygenated phospholipid species.

### CoQ_10_ extraction and LC-MS/MS analysis in cells.

To extract ubiquinones and their reduced forms from HAECs, the Folch procedure was used with slight modifications. The goal was to extract phospholipids and metabolites such as CoQ_10_ and their reduced forms, knowing that the reduced forms of ubiquinones are stable at acidic pH. Briefly, cells were resuspended in 0.5 mL of 0.75% KCl, followed by the addition of 0.3 mL of cold acidified methanol (MeOH/1% HCl/BHT). The samples were kept on ice and vortexed every 5 minutes for a total of 20 minutes. Then, 2.7 mL of cold chloroform and 1 mL of cold acidified methanol (MeOH/1% HCl/BHT) were added. The samples were kept on ice and vortexed every 10 minutes for a total of 60 minutes. After the first extraction, 3 mL of cold chloroform was added, and the extraction process was repeated. The lower CoQ-containing layer was separated by centrifugation (5 min, 17,000×g, 4°C) and then dried under a stream of nitrogen. The dried samples were resuspended in 2-propanol/acetonitrile/water (85/10/5) containing 5 mM ammonium formate and 0.1% formic acid, diluted if necessary, overlaid with nitrogen, and stored at −80°C until analysis. LC/ESI-MS analysis of CoQ_10_ and their reduced forms was performed on a Thermo Ultimate 3000 HPLC system coupled to a hybrid quadrupole-Orbitrap mass spectrometer (Q-Exactive, Thermo Fisher Scientific) with the Xcalibur operating system. The instrument was operated in positive ion mode at a voltage differential of −4.0 kV and a source temperature of 320°C. Sheath gas and S-lens were set at 20 and 65, respectively. The resolution was set at 140,000 with a scan range of m/z 150–1800 and a user-defined mass tolerance of 5 ppm. MS/MS was performed in data-dependent mode with HCD set at 24 and a resolution of 17,500. Metabolites were separated on a reverse-phase Accucore C30 column using the 150-minute gradient program described above. The internal standard (CoQ6) was added directly to the MS sample to a final concentration of 10 μM. Analytical data were acquired and analyzed using Xcalibur software. The precursor ions of CoQ_10_ and their reduced forms in positive ion mode were detected as the ammonium adducts [M+NH4]+. The structure of the identified metabolites was confirmed by fragmentation analysis.

### Reduction of CoQ_10_.

CoQ_10_ was reduced to ubiquinol (CoQ10-H2) using sodium borohydride [3]. All glassware was rinsed with hexane and dried under nitrogen prior to use. CoQ_10_ was initially dissolved in hexane to a concentration of 10 mM, then diluted 1:10 with hexane. Sodium borohydride (2 mg/100 μL) was added to the CoQ_10_ solution, followed by the addition of methanol to a final concentration of 5% (v/v). The mixture was vortexed for 5 minutes and incubated in the dark for 10 minutes. The reaction was quenched by adding an equal volume of acidified water, vortexing for 1 minute, and centrifuging at 1,500×g for 5 minutes at 4°C. The upper hexane layer, containing CoQ_10_H2, was transferred to a glass vial, overlaid with nitrogen, and stored at −80°C. CoQ_10_ and its reduced form then were used as reference standards to build the calibration curves.

### Integration of CoQ_10_ into liposomes for HAEC treatment.

To integrate CoQ_10_ (Sigma, C9538) into liposomes, a lipid film was first prepared using a 1:1 molar ratio of di-18:1-PC (DOPC) and CoQ_10_. The lipid and CoQ_10_ mixture was dried gently under a stream of 99.99% pure nitrogen gas to form a thin film. Following the formation of the lipid film, liposomes were prepared by rehydrating the film with phosphate-buffered saline, resulting in a final volume of 1 mL at a 1 mM concentration of liposomes containing CoQ_10_. For liposome formation, the dried lipid-CoQ_10_ film was sonicated in a sonication bath for 20 min to facilitate the integration of CoQ_10_ into the lipid bilayer. The solution was vortexed every 5 min for 30 sec during the sonication to ensure uniformity. A control group of liposomes without CoQ_10_ was also prepared using the same method for comparison in treatment assays. The resulting liposomes were used to treat HAECs, with the liposomes serving as a delivery vehicle to facilitate CoQ_10_ transfer into the cells.

### NADH consumption assay.

NADH consumption was measured by the change in Absorbance at 340 nm using 100 μl enzyme reactions in 100 mM Tris-HCl, pH 7.4 buffer in a 96-well microplate. Enzyme reactions contained 50 nM, 100 nM, 200 nM, or 500 nM of human recombinant CYB5R1 protein, 500 μM NADH (freshly prepared in Tris-HCl, pH 7.4) and 200 μM of different substrate candidates (CoQ0, a-tocopheryl quinone, glutathione disulfide (GSSG), Vitamin K2, dehydroascorbate). For experiments including CYB5B, CYB5R1:CYB5B was 1:1 (100 nM). A BioTek Microplate Reader was used to determine the absorbance at 340 nm every 1 min for 1 hr. Reactions without NADH or without enzyme were used for data normalization. Rates were calculated from the slopes of the initial linear reaction phase.

### DAB staining and transmission electron microscopy.

Transient expression of CYB5R1-APEXII was achieved by pCIGX-CYB5R1-APEX2 plasmid transfection into HAECs. To avoid arbitrary effects due to a high level of expression, we used a 0.20 μg/well of plasmid DNA to achieve expression levels roughly 2-fold above endogenous. HAECs were fixed with 2% (v/v) glutaraldehyde in 100 mM cacodylate solution for 1 hr at room temperature. Fixed cells were rinsed 3 times with 100 mM cacodylate solution and incubated with 20 mM glycine in 100 mM cacodylate solution for 5 min. Fixed cells were then washed 3 times with 100 mM cacodylate and loaded with 0.5 mg/ml 3,3′-Diaminobenzidine (DAB) and 1 mM H_2_O_2_. Once brown precipitates formed in CYB5R1-APEXII positive cells, cells were rinsed with 100 mM cacodylate solution 3 times and fixed in 2.5% glutaraldehyde prior to imaging. Cells were examined on a JEOL 1400 Plus transmission electron microscope with a side mount AMT 2k digital camera (Advanced Microscopy Techniques, Danvers, MA).

### Coumarin boronic acid (CBA) detection of hydrogen peroxide (H_2_O_2_) production.

Recombinant CYB5R1 was mixed with or without CoQ0 in 100 mM Tris-HCl pH 7.4 and transferred to a 96-well plate. CBA fluorescent probe and NADH were subsequently added to each well. Final reaction concentrations were as follows; 5 nM CYB5R1, 100 nM CoQ0, 500 μM NADH, 20 μM CBA. Hydrogen peroxide formation was monitored using a BioTek Microplate Reader to determine the fluorescence (350/450 nm) every 1 min at 37°C for 4 hr. The slope of the linear portion of the curve was used to calculate the rate of hydrogen peroxide production. For each group, negative controls were included by adding catalase to a final concentration of 500 μg/ml. The fluorescent signal from the corresponding catalase control was included, and the catalase-inhibitable amount was designated as H_2_O_2_.

### Seahorse XF analysis.

NT and CYB5R1 KD HAECs were plated at 150,000 cell/cm^2^ in a 96-well Seahorse XF96 extracellular analyzer plate. On the day of the experiment, the medium was aspirated, replaced with 180 μL DMEM, and allowed to incubate at 37°C (no CO_2_) for 1 hr. DMEM, oligomycin (2 μM), FCCP (0.5 μM), and rotenone (2 μM) were administered in sequence. Respiration (taken as Oxygen Consumption Rate, OCR) and Extracellular Acidification Rate (ECAR) were measured in sequence following injection of each drug, providing the following: basal respiration and ECAR, maximal respiration, total respiration, spare capacity for oxygen consumption, proton leak, and ATPase-driven respiration. Data were normalized to cell number, determined by crystal violet staining.

### Crystal violet staining.

The medium was aspirated gently, washed 1x with PBS, and fixed in 4% paraformaldehyde (PFA) for 10 min at room temperature. PFA was gently aspirated, replaced with 100 μL of crystal violet (0.1% dissolved in water), and allowed to incubate at room temperature for 30 min. The plate was then submerged in cold water several times until the water became clear. The plate was placed upside down and allowed to dry overnight. The following day, 100μL of 1% SDS was added to each well and allowed to shake for 30 min. Finally, 50 μL of sample was pipetted into a new 96-well plate and the absorbance at 550 nm was recorded. The absorbance at 550 nm is directly proportional to the number of live cells.

### Quantifying ATP with CellTiter-Glo luminescence.

NT and CYB5R1 KD HAECs were plated overnight in a black 96-well plate at a density of 10,000 cells/cm^2^. The next day, ATP standards were plated in 10-fold serial dilutions starting at 5 μM into empty wells. To deplete ATP, 1 μM Rotenone and 10 μM oligomycin were added to respective wells containing cells. The plate was incubated for 3 hr at 37°C/5% CO_2_. The plate was then allowed to equilibrate at room temperature for 30 min. Equal-volume CellTiter-Glo reagent (Promega, G7570) was added to each well and allowed to mix on an orbital shaker for 2 min at RTand the the luminescence signal recorded using a BioTek Microplate Reader (560 nm). Wells containing medium without cells were used to obtain a value for background luminescence.

### FerroOrange assay.

NT and CYB5R1 KD HAECs were trypsinized and seeded at 15,000 cells/cm^2^ in a 96-well plate in endothelial basal medium without supplementation. For the negative control condition, 100 μM iron chelator 2,2’-bipyridyl was added to the appropriate wells. For the positive control condition, 100 μM ferrous chloride was added to the appropriate wells. FerroOrange fluorescent probe (Dojindo, F374) was added to all wells at 1 μM and the plate was incubated at 37°C/5% CO_2_ for 30 min. Intracellular ferrous iron was measured by monitoring fluorescence (excitation: 543 nm; emission: 580 nm) using a BioTek Microplate Reader.

### Electron paramagnetic resonance.

EPR spectra of the Trolox phenoxyl radical, generated by the oxidation of Trolox with the soybean 15-LOX/AA complex in the presence or absence of CYB5R1/NADH/CoQ0, were recorded at 25°C in gas-permeable Teflon tubing (inner diameter: 0.8 mm; thickness: 0.013 mm; Alpha Wire) on a JES-FA 100 ESR spectrometer (JEOL, Kyoto, Japan) at X-band (9.4 GHz). The tubing (length: 20 cm) was filled with a 90-μL sample, double-folded, and placed in an open 3.0-mm (internal diameter) EPR quartz tube. Spectrometer settings were as follows: field center: 335.442 mT; microwave power: 10 mW; scan time: 30 s; time constant: 0.03 s; and modulation width: 0.12 mT. Briefly, the magnitude of the EPR signal of the Trolox phenoxyl radical was measured 1 min after the addition of 100 U/μL 15-LOX soybean to a 1 mM dispersion of arachidonic acid (AA) and 1 mM Trolox in buffer solution (25 mM phosphate buffer, pH 8, 100 μM DTPA). The reaction was initiated by adding 15-LOX to the mixture. The concentrations of CyB5R1, NADH, and CoQ0 were 5 nM, 100 μM, and 2 mM, respectively. In the experiment with varying NADH concentrations, the concentrations were 50 μM, 100 μM, and 200 μM.

### TNF-α stimulation in NT and CYB5R1 KD HAECs.

NT and CYB5R1 KD HAECs were seeded at 10,000 cells/cm^2^ in a 12-well plate and allowed to adhere overnight at 37°C/5% CO_2_. The following day, 10 ng/mL TNF-α of DMSO vehicle control were applied to the cells and incubated at 37°C/5% CO_2_ for 18 hr. HAECs were lysed directly in 2x Laemmli buffer and boiled at 95°C for 10 min prior to immunoblotting analysis. Rabbit VCAM-1 primary antibody and IR dye 800CW donkey anti-rabbit secondary antibody were used to assess endothelial inflammation. A mouse α-tubulin primary antibody (1:10000, MilliporeSigma, T9026) and IR dye 680RD donkey anti-mouse secondary antibody (1:15,000, Licor, 926–68072) was used as a loading control.

### TNF-α stimulation in NT and NRF2 KD HAECs.

NT or NRF2 KD HAECs were seeded at 10,000 cells/cm^2^ in a 12-well plate and allowed to adhere overnight at 37°C/5% CO_2_. The following day, 10 ng/mL TNF-α or DMSO vehicle control was applied simultaneously with CP50 (5 μM), NO_2_-OA (5 μM), or CDDO (50 nM) and incubated at 37°C/5% CO_2_ for 18 hr. HAECs were lysed directly in 2x Laemmli buffer and boiled at 95°C for 10 min prior to immunoblotting analysis.

### Generation of CYB5R1 knockout mice.

EC-specific CYB5R1 KO generation: the genome targeting vector for CYB5R1 was generated by the University of California (UC) Davis Knockout Mouse Repository (KOMP). Chimeras containing this targeting vector were crossed with FLPe recombinase mice to form CYB5R1^fl/fl^ mice. CYB5R1^fl/fl^ mice were crossed with Cdh5-CreER^T2^ mice and the resulting offspring were administered tamoxifen (10 mg/kg/day) for 10 days. This results in the excision of exon 3 of CYB5R1 solely in Cdh5 expressing cells resulting in EC-specific CYB5R1 knockout. Global CYB5R1 KO generation: cryopreserve sperm from C57BL/6N-*A*^*tm1Brd*^ *Cyb5r1*^*tm1a(KOMP)Wtsi*^/MbpMmucd strain (RRID:MMRRC_047271-UCD), was obtained from the Mutant Mouse Resource and Research Center (MMRRC) at University of California at Davis, an NIH-funded strain repository, and was donated to the MMRRC by The KOMP Repository, University of California, Davis; Originating from Kent Lloyd, UC Davis Mouse Biology Program (PMID: 21677750). The *Cyb5r1*^*tm1a(KOMP)Wtsi*^ is a reporter “KO first allele (reporter-tagged insertion with conditional potential)” allele and was recovered by In Vitro Fertilization (IVF) of eggs from C57BL6/J (Jackson Labs) donor females by the Mouse Embryos Services Facility (Department of Immunology, University of Pittsburgh). The KO first allele was converted to a conditional allele *Cyb5r1*^*tm1c (KOMP)Wtsi*^ by crossing the *Cyb5r1*^*tm1a(KOMP)Wtsi*^ strain with to a Flp Recombinase strain (B6.129S4-Gt(ROSA)26Sortm1(FLP1)Dym/RainJ).

### Human coronary collection and isolation.

Human coronary arteries were collected from subjects (Table 1) who provided consent and were enrolled in studies approved by the University of Pittsburgh’s Institutional Review Board (IRB) following the Declaration of Helsinki. Cadaveric donor tissues were obtained through the Center for Organ Recovery and Education (CORE) and sanctioned by the University of Pittsburgh Committee for Oversight of Research and Clinical Training Involving Decedents (CORID). A detailed protocol outlining tissue collection and handling has been published previously^42^. Personnel involved in specimen extraction and handling received extensive institutional training. Tissues were processed as close to the extraction time possible to ensure tissue integrity.

### Atherosclerosis animal experiments.

12-week-old C57BL/6J male mice were injected with AAV8-D377Y-mPCSK9 (5 × 10^9^ genome copies/gram body weight) and placed on a high-fat diet (Research Diets, D12492) on day 0. Blood was collected on day 0 to assess initial cholesterol levels. The mice remained on a high-fat diet for 16 weeks and blood was collected every 8 weeks. At week 16, mice were sacrificed, and tissue was collected for downstream processing. Oil red O staining was used to assess atherosclerotic lesions in aortic root cryosections. All animal studies were approved by the Institutional Animal Care and Use Committee (IACUC) at the University of Pittsburgh. All animals were housed in a pathogen free environment and supplied with standard drinking water and laboratory diet according to IACUC guidelines.

### Oil Red O staining.

Whole hearts were excised and fixed in 4% PFA for 24 hr at 4°C, then transferred to 30% sucrose for 24 hr at 4°C. Hearts were embedded in OCT and aortic root sections were obtained using a cryostat. The following procedure was conducted at room temperature. Heart sections were fixed in 4% PFA for 5 min, washed in 60% isopropyl alcohol for 5 min, and stained with filtered oil red-O (0.30% in isopropyl alcohol) for 10 min. Sections were then washed with 60% isopropyl alcohol for 2 min and rinsed quickly in water prior to Hematoxylin staining for 3 min. The hearts were washed in PBST (0.02% Tween 20 in PBS) 4 times, mounted with glass coverslips, and imaged with a brightfield microscope. Quantification of plaque area within the aortic root was performed in Fiji. Total plaque area was normalized to the total area of the aortic root.

### LC-MS analysis of cholesterol and cholesteryl esters.

For the quantification of cholesterol, cholesteryl esters, and triglycerides in plasma, 15 μL of each plasma sample was spiked with internal standards: 5 pmol of d_7_-cholesterol, 5 pmol of d_7_-16:0-cholesteryl ester, and 424 pmol of TG17:0/17:0/17:0 (Avanti Polar Lipids, Inc.) serving as internal standards for cholesterol, cholesteryl esters, and triglycerides, respectively. Lipid extraction was carried out by adding 400 μL of ethyl acetate, followed by 400 μL of water. After brief mixing and phase separation, the upper organic phase was collected, transferred to a clean glass vial, and evaporated to dryness under a stream of nitrogen. The dried lipid extract was reconstituted in 200 μL of a 1:1 (v/v) mixture of ethyl acetate and acetonitrile for subsequent analysis.

Chromatographic separation of cholesterol and cholesteryl esters was achieved using a C18 reverse-phase column (2 × 100 mm, 5 μm particle size; Phenomenex, Torrance, CA, USA) operated at a flow rate of 650 μL/min. The mobile phase consisted of solvent A (50% water/50% acetonitrile with 0.1% formic acid) and solvent B (90% isopropanol/10% acetonitrile with 0.1% formic acid). The elution gradient was initiated at 50% solvent B and linearly increased to 100% over 7.7 minutes. The column was held at 100% solvent B for 2 minutes, followed by re-equilibration to initial conditions over 3 minutes. Analyte quantification was performed using a QTrap 6500+ triple quadrupole mass spectrometer (Sciex, San Jose, CA, USA) equipped with an electrospray ionization (ESI) source operated in positive ion mode. Data acquisition was conducted in multiple reaction monitoring (MRM) mode. The monitored transitions were m/z 369.3 → 147.3 for cholesterol and cholesteryl esters, and m/z 376.3 → 147.3 for their deuterated analogs (d_7_-cholesterol and d_7_-cholesteryl ester). The ionization in the QTrap 6500+ source hydrolyzes cholesterol esters producing free cholesterol ions for quantification. The mass spectrometry parameters were set as follows: curtain gas at 40 units, ion source gas 1 at 55 units, ion source gas 2 at 50 units, ion spray voltage at 5500 V, and source temperature at 600 °C. The declustering potential was 90 eV, entrance potential was 5 eV, collision energy was 35 eV, and collision cell exit potential was 10 eV.

### LC-MS analysis of triglycerides.

Plasma triglycerides (TAG) were quantified and characterized using HPLC-HR-MS/MS. Separation was achieved on a C8 Luna column (2 × 150 mm, 5 μm, Phenomenex) at 0.4 ml/min with a gradient of acetonitrile/water (9:1, v/v) with 0.1% ammonium acetate (A) and isopropanol/acetonitrile (7:3, v/v) with 0.1% ammonium acetate (B): 35–100% B (0.1–10 min), 100% B (10–13 min), and 4 min re-equilibration. A Q-Exactive mass spectrometer (ThermoFisher) in positive mode was used with auxiliary gas heater at 250 °C, capillary at 300 °C, sheath gas flow 20, auxiliary gas flow 20, sweep gas flow 2, spray voltage 3 kV, and S-lens RF level 75%. Full MS scans (500–1100 m/z) were at 35000 resolution, and TAG characterization was performed by dd-MS2 of the top 5 peaks at 17500 resolution.

### LC-MS/MS Analysis of Ceramides.

LC-MS/MS analysis was performed using a Sciex QTRAP 6500 triple quadrupole mass spectrometer equipped with an electrospray ionization (ESI) source, coupled to a UHPLC system. Chromatographic separation was achieved using a Phenomenex reverse-phase C18 column (5 mM particle size, 100 × 2 mm; Phenomenex) maintained at 40°C. The mobile phases consisted of mobile phase A: 70:30 water/acetonitrile with 0.1% formic acid and mobile Phase B: 90:10 isopropanol/acetonitrile with 0.1% formic acid. The flow rate was set at 0.65 mL/min. The gradient started with 100% mobile phase A for 0.5 min, followed by a linear transition to 100% mobile phase B over 20 min, which was then held for an additional 10 min then coming back to starting conditions in the last 10 min. The mass spectrometer was operated in positive ion mode (ESI+) with the following parameters: Curtain Gas: 40, IonSpray Voltage: 5500 V, Source Temperature: 650°C, Collision Gas: Medium, Ion Source Gas 1: 60, Ion Source Gas 2: 45, Declustering Potential (DP): 80 V, Entrance Potential (EP): 10 V, Collision Energy (CE): 27 V, Collision Cell Exit Potential (CXP): 13 V. MRM transitions for ceramides were monitored following 264.4 Da, which corresponds to the sphingoid base backbone of ceramides. Data was analyzed using Analyst software (Sciex). Chromatographic peaks were manually integrated, and ceramides were expressed as the area ratio of each analyte relative to the cholesterol d-7 internal standard.

### Blood analysis.

Blood was collected from WT, global CYB5R1 KO, and ICDH5 cre (EC) CYB5R1 KO animals at the endpoint of the atherosclerosis study (16-weeks). Hematological parameters were measured using Heska (HemaTrue Inc.; Miami Lakes, FL), in accordance with manufacturer’s instructions.

### Data presentation and statistical analyses.

Data are presented as mean ± s.e.m. unless stated otherwise. For cell-based experiments, the graphs show the mean ± s.e.m. of n = x wells (x values are provided in the figure legends) representative of a single experiment, unless stated otherwise, performed independently y times (y value is provided in figure legends). Comparisons between groups were analyzed according to the statistical test specified in the figure legend. For the CP50 atherosclerosis study, an animal was omitted as determined by the ROUT outlier test. For LC-MS, statistical analyses were performed by one-way ANOVA, Tukey’s multiple comparisons test, unless otherwise specified. p < 0.05 was considered to be statically significant. Data were graphed using GraphPad Prism 10.2.3.

## Extended Data

## Figures and Tables

**Fig. 1:**
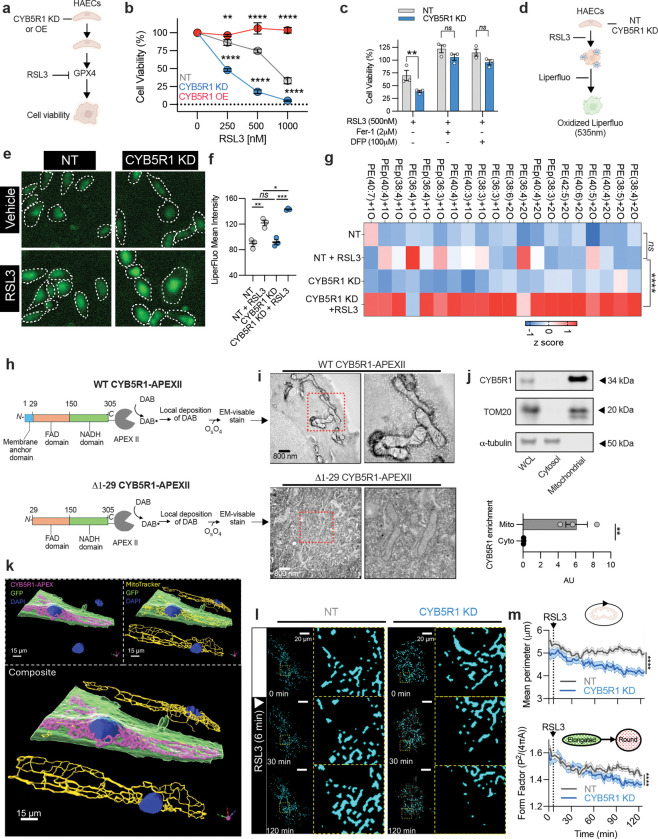
CYB5R1 is a potent ferroptosis suppressor located at the outer mitochondrial membrane. **a,** Schematic workflow of assessing cell viability using WST-8/PMS as an indicator of viable cells. **b,** Dose-dependent toxicity of RSL3-induced cell death of non-targeting (NT), CYB5R1 knockdown (KD), and CYB5R1 overexpressing (OE) HAECs. **c,** NT and CYB5R1 KD HAECs treated with 500 nM RSL3 with or without 2 μM ferrostatin-1 (Fer-1) or 100 μM deferiprone (DFP). **d,** Schematic workflow of assessing lipid peroxidation using liperfluo fluorescent probe. **e**, Epifluorescent microscopy of RSL3-induced (500 nM for 2 hr) liperfluo oxidation in NT and CYB5R1 KD cells. Images show one representative of three independently performed experiments. Images show n=7–9 representative cells from n=50–70 total cells. Cell outlines were overlayed as determined by the respective brightfield image. Cell outlines were overlayed as determined by the respective brightfield image. **f**, Quantification of liperfluo staining shown in **e**. Data are mean ± s.e.m. from one representative from three independently performed experiments. **g,** Heat-map showing the abundance of mono- and di-oxidized phospholipid species (PE, phosphotidylethanolamine) in NT and CYB5R1 KD HAECs treated with DMSO or RSL3. For the heat map, samples (*n* = 3) were averaged and normalized to cell number. Each lipid species was normalized to the maximum detected level. The experiment was performed independently twice. **h,** Schematic of determining WT CYB5R1-APEXII and Δ1–29 CYB5R1-APEXII localization via DAB staining and electron microscopy. **i,** Representative electron microscopy images from **h**. **j,** Representative immunoblots of naive HAECs subject to biochemical fractionation. Tubulin and TOM20 were used as positive controls for the cytosol and mitochondria, respectively. Immunoblot images are cropped from the IR signal files. Enrichment (below) was normalized to CYB5R1 in the whole cell lysate. Data show the averages from three independently peroformed experiments. **k,** Immunofluorescence imaging to assess CYB5R1 localization in HAECs. CYB5R1 was tethered to APEXII for antibody detection. GFP positive cells indicate a successfully transfected cell. MitoTracker Deep Red was used as a positive control for mitochondria. Image files were rendered to 3D using IMARIS software. **l,** Live-cell imaging of NT and CYB5R1 KD HAECs incubated with 1 μM RSL3 (added at 6 min) for 2 hr. MitoTracker Deep Red (cyan) was used to visualize mitochondria. **m**, Mitochondrial perimeter and form factor measured from **k**, calculated from 38 (NT) and 41 (CYB5R1 KD) individual mitochondria averaged from a single cell. Cell viability data are mean ± s.e.m. of n = 6 wells of a 96-well plate from one representative of three (**b**) independent experiments and n = 3 wells of a 96-well plate from the average of three (**c**) independent experiments, ****P < 0.0001, ***P < 0.001, **P < 0.01, ns is not significant; students t-test (**j**) and two-way ANOVA (**b,c,f,g,m**).

**Fig. 2:**
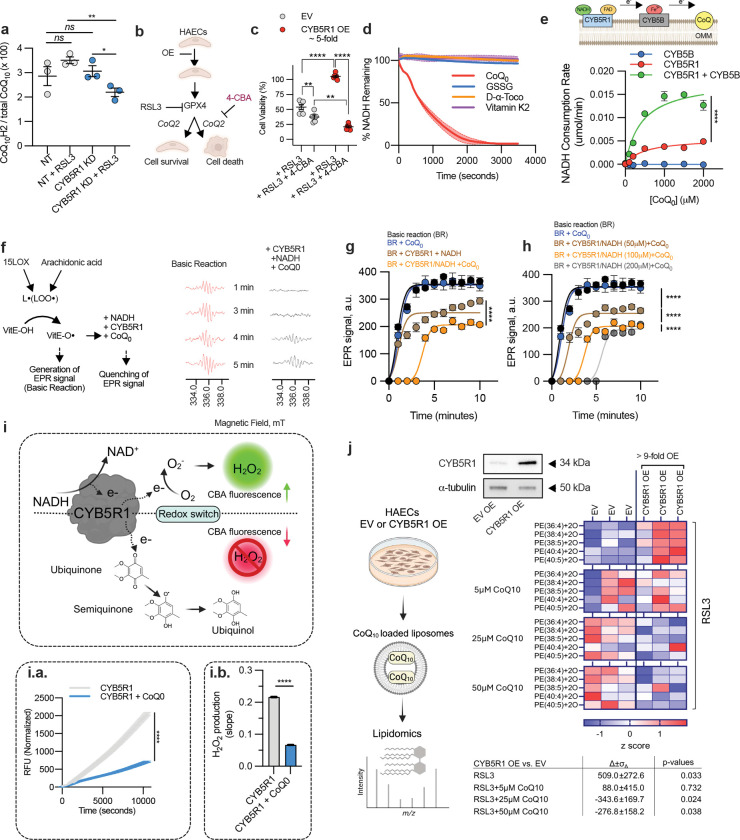
CYB5R1 reduces CoQ to suppress ferroptosis. **a,** Quantification of ubiquinol CoQ_10_ (CoQ_10_H2) relative to total CoQ_10_ in NT and CYB5R1 KD HAECs with or without 10 μM RSL3 treatment using liquid chromatography–mass spectrometry. Ubiquinone 6 was used as an internal standard. **b,** Schematic of depleting CoQ_10_ in HAECs by utilizing 4-CBA . **c,** RSL3-induced (500 nM) cell death of EV OE or CYB5R1 OE HAECs with or without 75 μM 4-CBA treatment for 48 hrs. Data are mean ± s.e.m. from n= 6 wells of a 96-well from one representative of three independent experiments. **d**, NADH consumption assay (340 nm) in tris buffer using recombinant CYB5R1 (100 nM) in combination with various electron acceptor molecules (2,3-dimethoxy-5-methyl-1,4-benzoquinone (CoQ_0_), oxidized glutathione (GSSG), D-α-Tocopheryl Quinone (Vitamin E), and Vitamin K_2_). Data are mean ± s.e.m. of three independently performed experiments. **e**, NADH consumption assay with increasing CoQ_0_ following the coincubation of recombinant CYB5R1 (Δ1–29) and CYB5B for 30 min at 37°C. The proposed mechanism is shown above. Data are mean ± s.e.m. of n = 3 wells of a 96-well plate from one representative of two independently performed experiments. A non-linear regression model was used to fit the data. **f**, Electron paramagnetic resonance (EPR) spectroscopy scheme of the enzymatic generation of Vitamin E (trolox) radical by 15LOX soybean and arachidonic acid (left). Representative EPR spectra for detecting Vitamin E radical formation of basic reaction (no CYB5R1/CoQ/NADH; left) and CYB5R1/CoQ/NADH (right) supplemented reactions are shown to the right. **g**, EPR assay to follow the production and quenching of vitamin E radical via CYB5R1 (100 μM NADH). Basic reaction (BR) is 15LOX soybean, arachadonic acid, and trolox. **h**, EPR assay as in **g** with varying [NADH]. **i**, Hydrogen peroxide (H_2_O_2_) generated by recombinant CYB5R1 (Δ1–29) with or without CoQ_0_ , as measured by coumarin boronate fluorescent probe (**i.a**). The production of H_2_O_2_ was quantified by measuring the slope of the linear segment of the curve (**i.b**). **j**, Schematic representation (left) and heat-map (right) showing the abundance of di-oxidized phospholipid species (PE, phosphotidylethanolamine) in EV OE and CYB5R1 OE (excess; >9-fold) HAECs treated with DMSO or RSL3 supplemented with CoQ_10_-integrated liposomes. For the heat map, samples (*n* = 3) were averaged and normalized to cell number. Each lipid species was normalized to the maximum detected level. The experiment was performed independently twice. Data are mean ± s.e.m. from n = 3–6 independent reactions (**g,h**) or n= 4 wells of a 96-well plate from one representative of three (**i**) independent experiments, ****P < 0.0001, ***P < 0.001, **P < 0.01,*P < 0.05; two-way ANOVA was used for all statistical analyses.

**Fig. 3:**
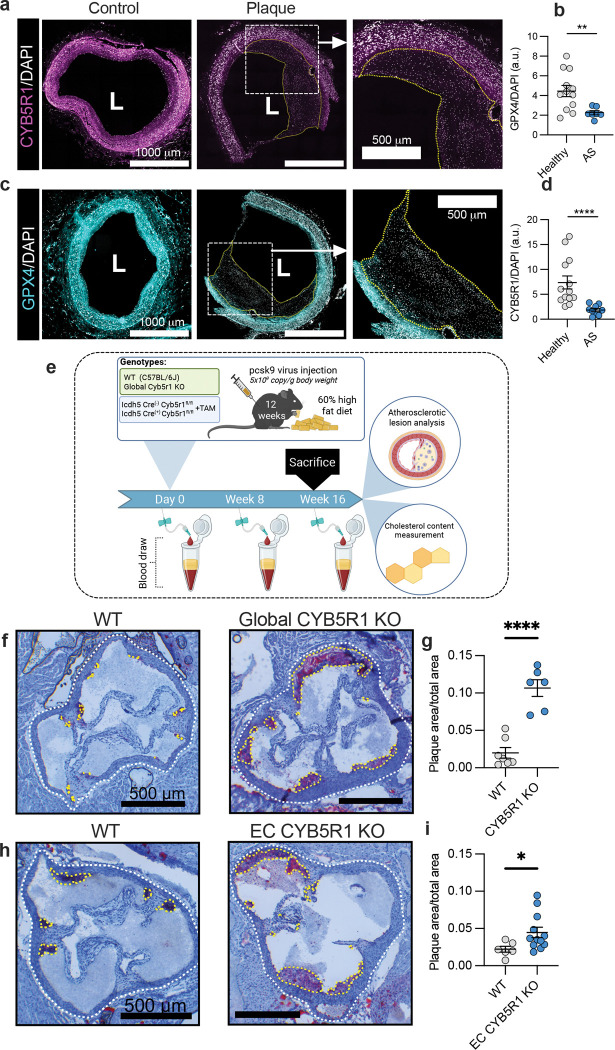
CYB5R1 KO mice develop severe atherosclerosis and CP50 reduces plaque burden **a-d**, Immunostaining of human coronary arteries in healthy and atherosclerotic human patients. CYB5R1 (**a**) and GPX4 (**c**) staining are shown in magenta and cyan, respectively. DAPI (white) was used as a nuclear marker. The quantifications (**b,d**) are the average from n=7–13 individual patients. Scale bars are 500 and 1000 μm. L=lumen. **e**, Experimental design of the atherosclerosis experiment with 12-week-old male C57BL/6J mice. The study was conducted with global CYB5R1 KO (n=6–7) and EC CYB5R1 KO (n=6–12; Icdh5 (PAC) Cre + CYB5R1fl/fl) mice. Blood was drawn every month to measure cholesterol content in the plasma. At the end of the study, mice were sacrificed, hearts excised and sectioned, and subject to Oil Red O staining. **f**, Representative Oil Red Ostaining images and of WT and global CYB5R1 KO aortic root cross-sections at the end of the experimental study. **g**, Quantification of atherosclerotic plaque area relative to total area from **f**. **h**, Representative Oil Red O staining images and of WT and EC CYB5R1 KO aortic root cross-sections at the end of the experimental study. **i**, Quantification of atherosclerotic plaque area relative to total area from **h**, ****P < 0.0001, **P <0.01, *P < 0.05, ns is not significant; Mann-Whitney U test (**b,d**) and student’s t-test (**g,i**).

**Fig. 4:**
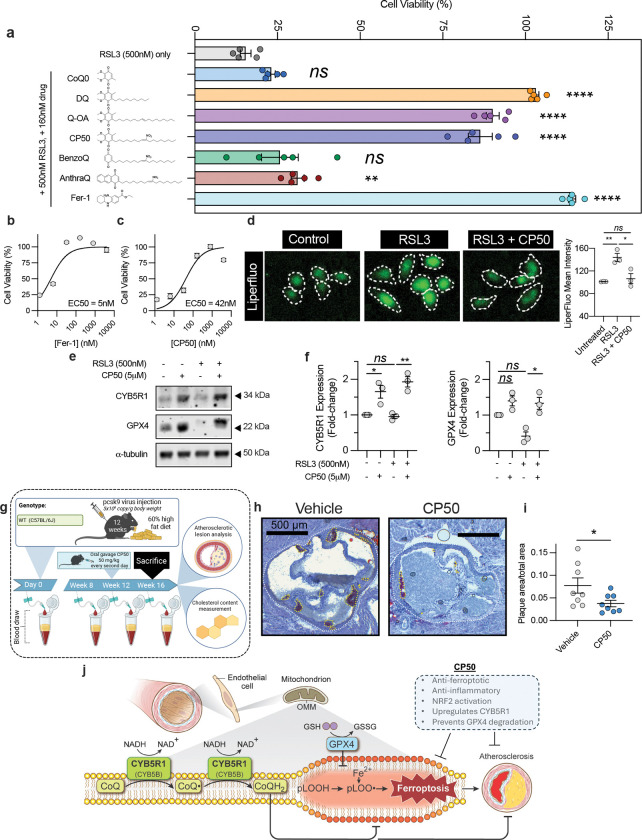
CP50 is a potent anti-ferroptotic molecule. **a,** HAECs treated with RSL3 (500nM) in combination with quinone derivatives (160nM) CoQ_0_, DQ (decylquinone-alkene), Q-OA (quinone-oleic acid), CP50, BenzoQ (benzoquinone-nitroalkene), and AnthraQ (anthraquinone-nitroalkene). Fer-1 was used as a positive control. The structure of each molecule is shown on the left. **b-c,** EC_50_ curves of Fer-1 (left) and CP50 (right) following treating HAECs with RSL3 (500nM). To calculate EC_50_ values, curves were fit to a non-linear regression model comparing response to drug concentration. **d**, Epifluorescent microscopy of RSL3-induced (500 nM for 2 hr) liperfluo oxidation in combination with CP50 (1μM) in HAECs. Images show one representative of three independently performed experiments. Each data point (right) represents n=40–70 cells of independent replicates from one representative of three independently performed experiments. **e,** Immunoblotting analysis of HAECs treated with RSL3 (500 nM) in combination with CP50 (5 μM) for 18 hr. CYB5R1 and GPX4 protein expression were normalized to α-tubulin. Immunoblot images are cropped from the IR signal files. The immunoblot is one representative of three independently performed experiments. **f**, Quantification of the immunoblot in **e**. The quantifications are the averages from three independent experiments. **g**, Experimental design of the atherosclerosis experiment with 12-week-old male C57BL/6J mice. The study was conducted with vehicle (labrasol, n=8) and CP50 (n=9) dosed mice. Blood was drawn every month to measure cholesterol content in the plasma. CP50 or labrasol (50 mg/kg) was administered at 12 weeks every other day for the final 4 weeks. At the end of the study, mice were sacrificed, hearts excised and sectioned, and subject to Oil Red O staining. **h**, Representative Oil Red O staining images and of vehicle and CP50 aortic root cross-sections at the end of the experimental study. **i**, Quantification of atherosclerotic plaque area relative to total area from **h**. **j**, Graphical illustration demonstrating the anti-ferroptotic function of CYB5R1. CYB5R1 forges an electron transfer relay with Coenzyme Q to suppress phospholipid peroxidation, preventing EC ferroptosis and AS pathogenesis. CYB5R1-mediated CoQ reduction can be accelerated by the hemoprotein CYB5B. The quinone-nitroalkene CP50 suppresses ferroptosis and AS pathogenesis through its unique ability to reduce oxidative stress and inflammation. Cell viability data are mean ± s.e.m. of n =5 (**a**,**b,c**) wells of a 96-well plate from one representative of two (**a,b,c**) independently performed experiments, ****P < 0.0001, ***P < 0.001, **P < 0.01,*P < 0.05, ns is not significant; student’s t-test (**i**), one-way ANOVA (**a,d**) and two-way ANOVA (**f**).

**Table 1:** 

Patient ID	Group	Age	Sex	Cause of Death	Hypertension	Diabetes	Smoking	Alcohol
2016-023-CORE	Plaque	49	Female	Stroke	Unknown	Unknown	Unknown	Yes
2016-034-CORE	Control	91	Male	Natural causes	No	No	No	Unknown
2016-037-CORE	Control	31	Male	Brain anoxia due to cardiovascular complications	No	No	Unknown	Unknown
2016-050-CORE	Control	65	Male	Stroke	HTN	No	Unknown	Yes
2016-061-CORE	Plaque	67	Male	Natural causes	No	Yes	Unknown	Unknown
2016-068-CORE	Control	68	Female	Intracranial hemorrhage	No	No	Yes	Unknown
2016-072-CORE	Control	62	Female	Cardiac arrest	No	No	No	Unknown
2016-091-CORE	Plaque	59	Female	Stroke	HTN	No	Unknown	Unknown
2016-092-CORE	Plaque	47	Female	Stroke	No	No	No	Unknown
2016-095-CORE	Plaque	87	Male	Myocardial Infarction	HTN	No	Yes	Unknown
2016-118-CORE	Plaque	62	Male	Cardiac arrest	No	No	No	Unknown
2016-168-CORE	Plaque	65	Male	Unknown	Yes	Yes	Unknown	Unknown
2016-174-CORE	Control	56	Female	Accident	Unknown	Unknown	Unknown	Unknown
2016-177-CORE	Control	22	Male	Motor accident	No	No	Unknown	Unknown
2017-015-CORE	Control	13	Male	Head trauma	Unknown	Unknown	Unknown	Unknown
2017-035-CORE	Control	48	Female	Brain anoxia due CO poisoning	Unknown	Unknown	Unknown	Unknown
2017-043-CORE	Control	57	Male	Heart attack	Unknown	Unknown	Unknown	Unknown
2017-044-CORE	Plaque	87	Female	Heart attack	Yes	Unknown	Yes	Unknown
2017-062-CORE	Plaque	66	Female	Scieroderma	Unknown	Unknown	Unknown	Unknown

HTN = Hypertension

## References

[1] Pahwa R. & Jialal I. in StatPearls (StatPearls Publishing, 2025).

[2] Libby P. Atherosclerosis. Nat. Rev. Dis. Primers 5, 56 (2019). 31420554 10.1038/s41572-019-0106-z

[3] Batty M., Bennett M. R. & Yu E. The role of oxidative stress in atherosclerosis. Cells 11, (2022). 10.3390/cells11233843 PMC9735601 36497101

[4] Jiang X., Stockwell B. R. & Conrad M. Ferroptosis: mechanisms, biology and role in disease. Nat. Rev. Mol. Cell Biol. 22, 266–282 (2021). 33495651 10.1038/s41580-020-00324-8 PMC8142022

[5] Ouyang S. Ferroptosis: the potential value target in atherosclerosis. Cell Death Dis. 12, 782 (2021). 34376636 10.1038/s41419-021-04054-3 PMC8355346

[6] Yan H.-F. Ferroptosis: mechanisms and links with diseases. Signal Transduct. Target. Ther. 6, 49 (2021). 33536413 10.1038/s41392-020-00428-9 PMC7858612

[7] Feng S. The mechanism of ferroptosis and its related diseases. Mol. Biomed. 4, 33 (2023). 37840106 10.1186/s43556-023-00142-2 PMC10577123

[8] Han C. Ferroptosis and its potential role in human diseases. Front. Pharmacol. 11, 239 (2020). 32256352 10.3389/fphar.2020.00239 PMC7090218

[9] Gao R. FSP1-mediated ferroptosis in cancer: from mechanisms to therapeutic applications. Apoptosis 29, 1019–1037 (2024). 38615304 10.1007/s10495-024-01966-1

[10] Li W., Liang L., Liu S., Yi H. & Zhou Y. FSP1: a key regulator of ferroptosis. Trends Mol. Med. 29, 753–764 (2023). 37357101 10.1016/j.molmed.2023.05.013

[11] Yang M. Ferroptosis of macrophages facilitates bone loss in apical periodontitis via NRF2/FSP1/ROS pathway. Free Radic. Biol. Med. 208, 334–347 (2023). 37619958 10.1016/j.freeradbiomed.2023.08.020

[12] Hall R., Yuan S., Wood K., Katona M. & Straub A. C. Cytochrome b5 reductases: Redox regulators of cell homeostasis. J. Biol. Chem. 298, 102654 (2022). 36441026 10.1016/j.jbc.2022.102654 PMC9706631

[13] Sui X. RSL3 drives ferroptosis through GPX4 inactivation and ROS production in colorectal cancer. Front. Pharmacol. 9, 1371 (2018). 30524291 10.3389/fphar.2018.01371 PMC6262051

[14] Wang C. Dual degradation mechanism of GPX4 degrader in induction of ferroptosis exerting anti-resistant tumor effect. Eur. J. Med. Chem. 247, 115072 (2023). 36603510 10.1016/j.ejmech.2022.115072

[15] Chen X., Yu C., Kang R. & Tang D. Iron metabolism in ferroptosis. Front. Cell Dev. Biol. 8, 590226 (2020). 33117818 10.3389/fcell.2020.590226 PMC7575751

[16] Zhao Y. The role of erastin in ferroptosis and its prospects in cancer therapy. Onco. Targets. Ther. 13, 5429–5441 (2020). 32606760 10.2147/OTT.S254995 PMC7295539

[17] Kagan V. E. Oxidized arachidonic and adrenic PEs navigate cells to ferroptosis. Nat. Chem. Biol. 13, 81–90 (2017). 27842066 10.1038/nchembio.2238 PMC5506843

[18] Yang W. S. & Stockwell B. R. Ferroptosis: death by lipid peroxidation. Trends Cell Biol. 26, 165–176 (2016). 26653790 10.1016/j.tcb.2015.10.014 PMC4764384

[19] Feng H. & Stockwell B. R. Unsolved mysteries: How does lipid peroxidation cause ferroptosis? PLoS Biol. 16, e2006203 (2018). 29795546 10.1371/journal.pbio.2006203 PMC5991413

[20] Martell J. D., Deerinck T. J., Lam S. S., Ellisman M. H. & Ting A. Y. Electron microscopy using the genetically encoded APEX2 tag in cultured mammalian cells. Nat. Protoc. 12, 1792–1816 (2017). 28796234 10.1038/nprot.2017.065 PMC5851282

[21] Bersuker K. The CoQ oxidoreductase FSP1 acts parallel to GPX4 to inhibit ferroptosis. Nature 575, 688–692 (2019). 31634900 10.1038/s41586-019-1705-2 PMC6883167

[22] Doll S. FSP1 is a glutathione-independent ferroptosis suppressor. Nature 575, 693–698 (2019). 31634899 10.1038/s41586-019-1707-0

[23] Liang D. & Jiang X. START smuggling CoQ to fight ferroptosis. Nat. Cell Biol. 25, 207–208 (2023). 36658221 10.1038/s41556-022-01044-1

[24] Deshwal S. Mitochondria regulate intracellular coenzyme Q transport and ferroptotic resistance via STARD7. Nat. Cell Biol. 25, 246–257 (2023). 36658222 10.1038/s41556-022-01071-y PMC9928583

[25] Ma M.-Y. Defects in CYB5A and CYB5B impact sterol-C4 oxidation in cholesterol biosynthesis and demonstrate regulatory roles of dimethyl sterols. Cell Rep. 43, 114912 (2024). 39489939 10.1016/j.celrep.2024.114912

[26] Kagan V. E., Fabisiak J. P. & Quinn P. J. Coenzyme Q and vitamin E need each other as antioxidants. Protoplasma 214, 11–18 (2000).

[27] Yan B. Membrane Damage during Ferroptosis Is Caused by Oxidation of Phospholipids Catalyzed by the Oxidoreductases POR and CYB5R1. Mol. Cell 81, 355–369.e10 (2021). 33321093 10.1016/j.molcel.2020.11.024

[28] Xu X. The mechanisms of ferroptosis and its role in atherosclerosis. Biomed. Pharmacother. 171, 116112 (2024). 38171246 10.1016/j.biopha.2023.116112

[29] Kansanen E. Nrf2-dependent and -independent responses to nitro-fatty acids in human endothelial cells: identification of heat shock response as the major pathway activated by nitro-oleic acid. J. Biol. Chem. 284, 33233–33241 (2009). 19808663 10.1074/jbc.M109.064873 PMC2785166

[30] Khoo N. K. H., Li L., Salvatore S. R., Schopfer F. J. & Freeman B. A. Electrophilic fatty acid nitroalkenes regulate Nrf2 and NF-κB signaling:A medicinal chemistry investigation of structure-function relationships. Sci. Rep. 8, 2295 (2018). 29396403 10.1038/s41598-018-20460-8 PMC5797128

[31] Yuan S. & Straub A. C. STING inhibition enables efficient plasmid-based gene expression in primary vascular cells: A simple and cost-effective transfection protocol. PLoS One 19, e0303472 (2024). 38990864 10.1371/journal.pone.0303472 PMC11238992

[32] Xue P., Crum C. M. & Thibodeau P. H. Regulation of ABCC6 trafficking and stability by a conserved C-terminal PDZ-like sequence. PLoS One 9, e97360 (2014). 24840500 10.1371/journal.pone.0097360 PMC4026322

[33] Yuan S. Cooperation between CYB5R3 and NOX4 via coenzyme Q mitigates endothelial inflammation. BioRxiv (2021). doi:10.1101/2021.08.12.456058 PMC8577475 34656824

[34] Kagan V. E. Redox epiphospholipidome in programmed cell death signaling: catalytic mechanisms and regulation. Front. Endocrinol. (Lausanne) 11, 628079 (2020). 33679610 10.3389/fendo.2020.628079 PMC7933662

[35] Kapralov A. A. Redox lipid reprogramming commands susceptibility of macrophages and microglia to ferroptotic death. Nat. Chem. Biol. 16, 278–290 (2020). 32080625 10.1038/s41589-019-0462-8 PMC7233108

[36] Dosunmu-Ogunbi A. Endothelial superoxide dismutase 2 is decreased in sickle cell disease and regulates fibronectin processing. Function (Oxf) 3, zqac005 (2022). 35274104 10.1093/function/zqac005 PMC8900267

[37] Kagan V. E. & Gorbunov N. V. EPR measurements of nitric oxide-induced chromanoxyl radicals of vitamin E. Interactions with vitamin C. Methods Mol. Biol. 108, 277–284 (1998). 9921537 10.1385/0-89603-472-0:277

[38] Stoyanovosky D. A. Detection and characterization of the electron paramagnetic resonance-silent glutathionyl-5,5-dimethyl-1-pyrroline N-oxide adduct derived from redox cycling of phenoxyl radicals in model systems and HL-60 cells. Arch. Biochem. Biophys. 330, 3–11 (1996). 8651701 10.1006/abbi.1996.0219

[39] Kumar S., Kang D.-W., Rezvan A. & Jo H. Accelerated atherosclerosis development in C57Bl6 mice by overexpressing AAV-mediated PCSK9 and partial carotid ligation. Lab. Invest. 97, 935–945 (2017). 28504688 10.1038/labinvest.2017.47 PMC5563968

[40] Goettsch C. A single injection of gain-of-function mutant PCSK9 adeno-associated virus vector induces cardiovascular calcification in mice with no genetic modification. Atherosclerosis 251, 109–118 (2016). 27318830 10.1016/j.atherosclerosis.2016.06.011 PMC4983246

[41] Keeter W. C., Carter N. M., Nadler J. L. & Galkina E. V. The AAV-PCSK9 murine model of atherosclerosis and metabolic dysfunction. Eur. Heart J. Open 2, oeac028 (2022). 35919346 10.1093/ehjopen/oeac028 PMC9242032

[42] Cuevas R. A. Isolation of Human Primary Valve Cells for In vitro Disease Modeling. J. Vis. Exp. (2021). doi:10.3791/62439 PMC8406428 33938898

